# Non‐redundant functions of H2A.Z.1 and H2A.Z.2 in chromosome segregation and cell cycle progression

**DOI:** 10.15252/embr.202052061

**Published:** 2021-08-23

**Authors:** Raquel Sales‐Gil, Dorothee C Kommer, Ines J de Castro, Hasnat A Amin, Veronica Vinciotti, Cristina Sisu, Paola Vagnarelli

**Affiliations:** ^1^ College of Health, Medicine and Life Science Brunel University London London UK; ^2^ College of Engineering, Design and Physical Sciences Research Institute for Environment Health and Society Brunel University London London UK; ^3^ Present address: Department of Infectious Diseases Integrative Virology Heidelberg University Hospital Heidelberg Germany; ^4^ Present address: Department of Mathematics University of Trento Trento Italy

**Keywords:** centromere, CPC, MYC, senescence, spindle assembly checkpoint, Cell Cycle, Chromatin, Transcription & Genomics

## Abstract

H2A.Z is a H2A‐type histone variant essential for many aspects of cell biology, ranging from gene expression to genome stability. From deuterostomes, H2A.Z evolved into two paralogues, H2A.Z.1 and H2A.Z.2, that differ by only three amino acids and are encoded by different genes (*H2AFZ* and *H2AFV*, respectively). Despite the importance of this histone variant in development and cellular homeostasis, very little is known about the individual functions of each paralogue in mammals. Here, we have investigated the distinct roles of the two paralogues in cell cycle regulation and unveiled non‐redundant functions for H2A.Z.1 and H2A.Z.2 in cell division. Our findings show that H2A.Z.1 regulates the expression of cell cycle genes such as Myc and Ki‐67 and its depletion leads to a G1 arrest and cellular senescence. On the contrary, H2A.Z.2, in a transcription‐independent manner, is essential for centromere integrity and sister chromatid cohesion regulation, thus playing a key role in chromosome segregation.

## Graphical abstract

This study shows that the very similar histone variants H2A.Z.1 and H2A.Z.2 have different functions in chromatin organisation and cell cycle regulation.

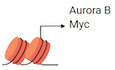

## Introduction

Nucleosomes form the basic unit of eukaryotic chromatin and consist of 146 DNA base pairs wrapped around an octamer of histone proteins. Canonical histones are incorporated into nucleosomes during DNA replication but histone variants, encoded by separate genes, are typically incorporated throughout the cell cycle (Filipescu *et al*, 2013; Skene & Henikoff, 2013; Turinetto & Giachino, 2015).

H2A is one of the four core histones. Sequence analyses have shown large‐scale divergence in the H2A family, resulting in numerous variants (Bonisch & Hake, 2012) including H2A.Z, a highly conserved variant originally identified in mouse cells (West & Bonner, 1980). H2A.Z is present as a single variant until early deuterostomes when two H2A.Z paralogues appear: H2A.Z.1 and H2A.Z.2; they differ by only three amino acids and are encoded by the *H2AFZ* and *H2AFV* genes, respectively (Coon *et al*, 2005). H2A.Z.1 and H2A.Z.2 present a different L1 loop structure, and studies using fluorescence recovery after photobleaching (FRAP) showed that H2A.Z.2‐containing nucleosomes are more stable than the H2A.Z.1‐containing ones (Horikoshi *et al*, 2013). In primates, H2A.Z.2 has also two splice variants: H2A.Z.2.1 and H2A.Z.2.2, where H2A.Z.2.2 has a shorter docking domain and forms highly unstable nucleosomes (Bonisch *et al*, 2012).

Although H2A.Z knockdown leads to early embryonic lethality in Drosophila (van Daal & Elgin, 1992) and mice (Faast *et al*, 2001), depletion of the H2A.Z orthologue in *S. cerevisiae*, HTZ1, is not lethal (Jackson & Gorovsky, 2000), indicating a possible difference in the role of H2A.Z among species.

Several studies have highlighted the importance of H2A.Z in transcription regulation (Gevry *et al*, 2007; Giaimo *et al*, 2018; Dalvai *et al*, 2013; Chevillard‐Briet *et al*, 2014; Rispal *et al*, 2019). However, whether H2A.Z promotes or represses transcription appears to depend on the gene, chromatin complex and post‐translational modifications of H2A.Z itself (Bargaje *et al*, 2012; Procida *et al*, 2021). In several organisms, H2A.Z peaks at transcriptional start sites (TSS) of active and repressed genes (Guillemette *et al*, 2005; Li *et al*, 2005; Raisner *et al*, 2005; Whittle *et al*, 2008) and localises at the +1 nucleosomes in the direction of transcription (Bagchi *et al*, 2020). H2A.Z has also been linked to heterochromatin regulation: recent evidence suggests that H2A.Z and H3K9me3, a known marker for heterochromatin, can cooperate to enhance the binding of Heterochromatin Protein 1 alpha (HP1α) to chromatin *in vitro* (Whittle *et al*, 2008; Ryan & Tremethick, 2018; Fan *et al*, 2004).

However, the specific contribution of each paralogue towards these quite different aspects of chromatin biology is currently not clear. Although H2A.Z.1 and H2A.Z.2 are distributed similarly in the nucleus and are subjected to comparable post‐translational modifications, their 3D structure and tissue distribution appear to be quite different (Dryhurst *et al*, 2009; Horikoshi *et al*, 2013). In fact, H2A.Z.2 does not compensate for the loss of H2A.Z.1 *in vivo*, as H2A.Z.1 knock‐out is lethal in mouse (Faast *et al*, 2001).

To date, very few studies have attempted to investigate and differentiate the specific roles of these two paralogues in vertebrates. In chicken DT40 cells, knock‐out of H2A.Z.2 results in a slower cell proliferation rate compared with the wild‐type and H2A.Z.1 knock‐out cells (Matsuda *et al*, 2010), while in humans, the Floating‐Harbor syndrome (Greenberg *et al*, 2019) and malignant melanoma (Vardabasso *et al*, 2015) have been specifically linked to H2A.Z.2 (Greenberg *et al*, 2019). In addition, both variants seem to play independent roles in the transcription of genes involved in the response to neuronal activity (Dunn *et al*, 2017). Moreover, very recently, it was shown that the differences between H2A.Z.1 and H2A.Z.2 on transcription regulation seem to depend more on the relative level of the two paralogues rather than on their chromatin localisation (Lamaa *et al*, 2020). However, we are still missing a full understanding of the role of each variant in human cells.

As the importance of histone variants in genome organisation and regulation becomes more appreciated and a few studies have linked H2A.Z to cancer, it is important to investigate and clarify the possible differential roles of the H2A.Z paralogues and their splice variants in cell cycle regulation *in vivo*. This will not only provide a better understanding of their function, but it will also reveal their relative contribution to divergent aspects of chromatin biology. In this study, we have used siRNA to specifically knockdown H2A.Z.1 or H2A.Z.2 in human cells. Our results show for the first time that H2A.Z.1 and H2A.Z.2 perform non‐redundant roles during cell cycle: whereas H2A.Z.1 is a key regulator of cell cycle progression at the G1/S boundary, H2A.Z.2 controls chromosome segregation and the spindle checkpoint function at the M/G1 transition.

## Results

### H2A.Z.2 is essential for genome stability

The H2A.Z histone variant has been linked to several and diverse functions in cellular biology and homeostasis. However, recent work has highlighted that the two H2A.Z paralogues, H2A.Z.1 and H2A.Z.2, do not perform completely overlapping roles. We therefore set out to investigate whether a separation of functions between the two paralogues was occurring in cell cycle regulation.

**Figure EV1:**
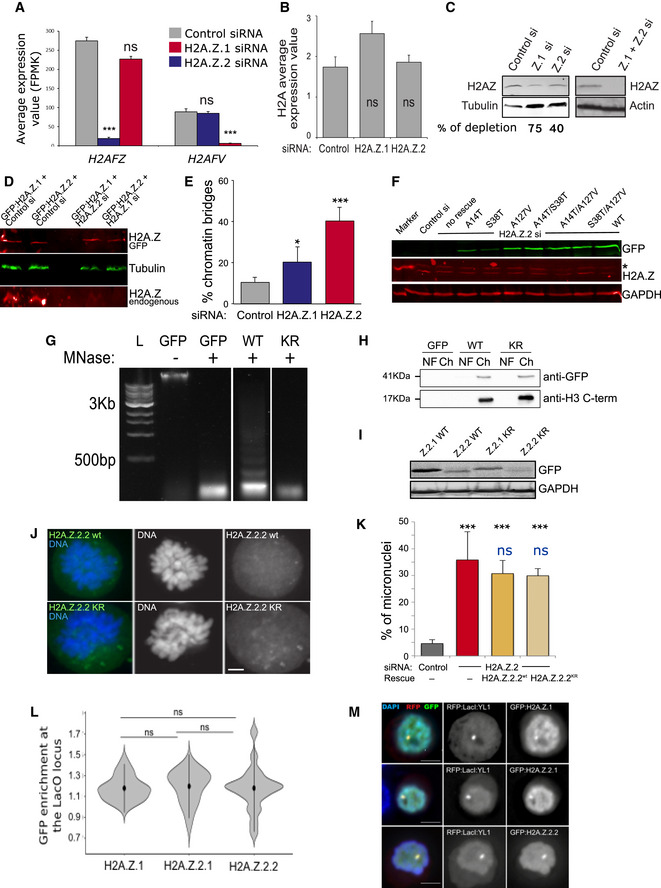
H2A.Z.2.1 knockdown leads to genome instability *H2AFZ* and *H2AFV* average expression values obtained by RNA sequencing of three biological replicates after control, H2A.Z.1 and H2A.Z.2 siRNA treatment. Error bars show the standard deviation (SD). ***P < 0.001; ns, not significant.
*H2A* average expression values obtained by RNA sequencing of three biological replicates after control H2A.Z.1 or H2A.Z.2 siRNA treatment. Error bars show the standard deviation (SD). ns, not significant.Western blot of HeLa whole cell lysates after control, H2A.Z.1, H2A.Z.2 or H2A.Z.1 + H2.A.Z.2 siRNA and probed with anti H2AZ antibody or actin or tubulin. The single depletions were imaged and quantified by LICOR.Western blot of HeLa cells transfected with control si + GFP:H2A.Z.1, with control si + GFP:H2A.Z.2, H2A.Z.1 si + GFP:H2A.Z.2 and H2A.Z.2 si + GFP:H2A.Z.1. The blot was probed with H2A.Z (red) and tubulin (green) antibodies and imaged by LICOR. Top panel shows the GFP:H2A.Z and the bottom panel the endogenous H2A.Z.Quantification of the number of anaphases with chromatin bridges of HeLa cells after transfection with control, H2A.Z.1 or H2A.Z.2 siRNA. Error bars represent SD of three biological replicates. At least 100 anaphases were analysed for each condition. Data sets were statistically analysed using Chi‐square test. *P < 0.05; ***P < 0.001.Western blot analysis using an anti‐GFP, H2A.Z and GAPDH antibodies of HeLa cells transfected with H2A.Z.2 siRNA and the indicated GFP constructs. The blot was imaged by LICOR. The star indicates a non‐specific band.HeLa cells were transfected with GFP, GFP:H2A.Z.2.1WT (WT) or GFP:H2A.Z.2.1KR (KR), lysed and digested with micrococcal nuclease (MNase) for 30 min to generate mononucleosomes. L: DNA ladder.The chromatin fraction (Ch) from (I) was separated on SDS–PAGE, together with the nuclear fraction (NF), and subjected to GFP immunoblotting. Anti‐H3 C‐terminus antibody was used as a control.Western blot analysis using anti‐GFP antibody in cells transfected with H2A.Z.2 siRNA and each of the indicated GFP constructs.Representative images of prometaphase chromosomes from HeLa cells co‐transfected with H2A.Z.2 siRNA and either H2A.Z.2wt or H2A.Z.2KR mutant Scale bar: 5 μm.Quantification of the percentage of cells with micronuclei from experiment (J). The error bars represent the SD of three biological replicates (control si *N* = 887; H2A.Z.2 si *N* = 1,005; H2A.Z.2 si + H2A.Z.2.2wt *N* = 465; H2A.Z.2si + KR *N* = 353). Data sets were statistically analysed using Chi‐square test: ***P < 0.001; ns, not significant. Black refers to the comparison with the control RNAi, and blue refers to the comparison with the H2A.Z.2si + H2A.Z.2.2wt data.GFP enrichment was calculated as a ratio between the intensity at LacI spot and the mean of two random nuclear spots. Mean and SD are shown. Data sets were statistically analysed using Wilcoxon rank test. ns, not significant.Representative images of DT40 cells carrying a LacO array inserted at a single locus co‐transfected with RFP:LacI:YL1 (red) and either GFP:H2A.Z.1, GFP:H2A.Z.2.1 or GFP:H2A.Z.2.2 (green). Scale bar: 5 μm.

To this purpose, we used RNA interference to specifically deplete each variant in HeLa cells. We conducted RNA‐seq on the control, H2A.Z.1 and H2A.Z.2 siRNA‐treated HeLa cells to confirm the specificity of the depletion. The data show that: (i) the siRNAs are specific for each paralogue; (ii) in HeLa cells, as in many other systems, H2A.Z.1 is expressed at a much higher level than H2A.Z.2; (iii) the removal of one form does not interfere with the expression level of the other (Fig EV1A and C, and D) or histone H2A (Fig EV1B). Since there are no antibodies that can specifically distinguish between the two paralogues, we checked that the double depletion was indeed effective in depleting both forms by immunoblotting (Fig EV1C). We also analysed the percentage of depletion contributed by each single variant siRNA against the pool of H2A.Z. As the RNA‐seq data suggested, the Western blot analyses confirmed that the major pool of H2A.Z in HeLa cells is provided by the H2A.Z.1 variant (Fig EV1C).

**Figure 1:**
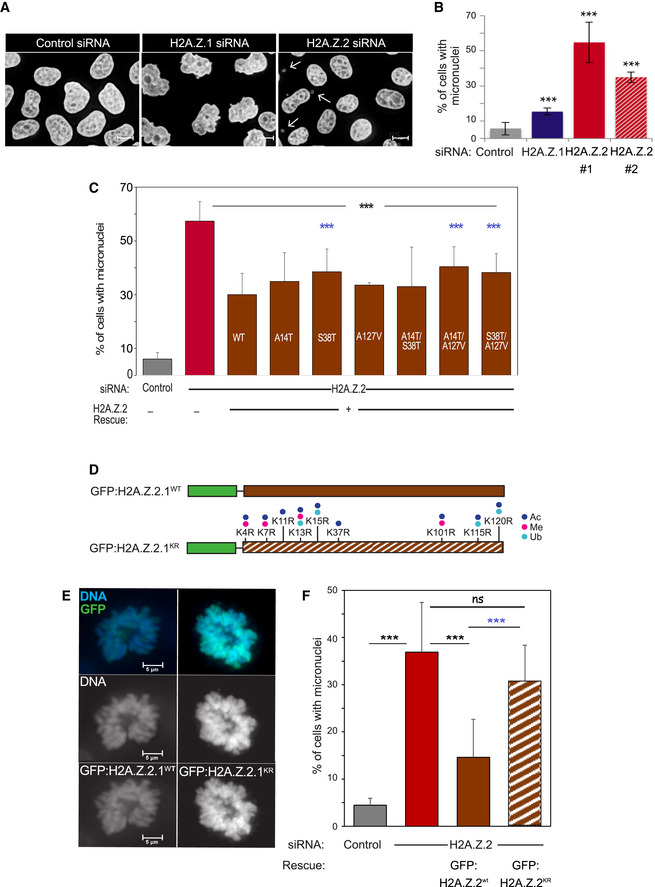
H2A.Z.2.1 knockdown leads to genome instability Representative images of HeLa cells treated with control, H2A.Z.1 or H2A.Z.2 siRNA for 72 h, fixed and stained with DAPI. White arrows point at micronuclei. Scale bar: 10 μm.Quantification of the percentage of cells with micronuclei from experiment in (A). Three biological replicates were analysed for each condition (control si *N* = 1,271; H2A.Z.1 si *N* = 1,091; H2A.Z.2#1 si *N* = 1,188; H2A.Z.2#2 si *N* = 1,193). The error bars represent the SD. Data sets were statistically analysed using Chi‐square test. ***P < 0.001.Quantification of the percentage of cells with micronuclei from HeLa cells co‐transfected with H2A.Z.2 siRNA and GFP:H2A.Z.2.1^WT^ with either single or double mutations. Error bars represent the SD of three biological replicates (control si *N* = 1,316; H2A.Z.2 si *N* = 1,877; H2A.Z.2 si + wt *N* = 1,973; H2A.Z.2 si + A14T *N* = 618; H2A.Z.2 si + S38T *N* = 558; H2A.Z.2 si + A127V *N* = 649; H2A.Z.2 si + A14T/S38T *N* = 1,090; H2A.Z.2 si + A14T/A127V *N* = 1,038; H2A.Z.2 si + S38T/A127V *N* = 1,150). Data sets were statistically analysed using Chi‐square test. ***P < 0.001; black stars refer to the comparison with the H2A.Z.2si data, and blue stars refer to the comparison with the H2A.Z.2si + wt data; no stars = non‐significant.Schemes of the GFP constructs used for the rescue experiments in (E) and (F). Green boxes represent the GFP, brown boxes represent the H2A.Z.2.1 isoform. Solid fill represents the WT construct whereas striped box represents the KR mutant form where the residues indicate the mutations performed. Dots represent the possible post‐translational modifications in the mutated amino acids: acetylation (ac, dark blue), methylation (me, pink) and ubiquitination (ub, light blue).Representative images of prometaphase chromosomes from HeLa cells co‐transfected with H2A.Z.2 siRNA and each of the constructs in (D) (green). Scale bar: 5 μm.Quantification of the percentage of cells with micronuclei from experiment (E). The error bars represent the SD of three biological replicates (control si *N* = 887; H2A.Z.2 si *N* = 1,005; H2A.Z.2 si + wt *N* = 760; H2A.Z.2 si + KR *N* = 479). Data sets were statistically analysed using Chi‐square test. ***P < 0.001; ns, not significant. Blue stars refer to the comparison with the H2A.Z.2 si + wt data.

We then moved to characterise cell division and cell cycle progression in the absence of each paralogue. H2A.Z.2‐depleted cells presented a high incidence of micronuclei, small nuclei formed when a chromosome or a fragment of a chromosome fails to be incorporated into the main cell nucleus after mitosis (Fig 1A—white arrows). We quantified the percentage of cells with micronuclei (Fig 1B) and the percentage of anaphase cells with chromatin bridges or lagging chromatin (Fig EV1E) upon depletion of each variant: although both H2A.Z.1 and H2A.Z.2 depletion increased the number of micronuclei and anaphase bridges, the phenotype was much stronger in H2A.Z.2‐depleted cells. This effect was also confirmed using an independent siRNA oligo against H2A.Z.2 (H2A.Z.2 #2) (Fig 1B). As H2A.Z.1 and H2A.Z.2 only differ by three amino acids, we also asked whether a particular amino acid was crucial for H2A.Z.2 role in genome stability. To this purpose, we mutated each of the three distinct amino acids of H2A.Z.2 back to the one present in H2A.Z.1, either individually or in combination, and performed rescue experiments. The H2A.Z.2 siRNA oligo targets the mRNA 5’ UTR, and therefore, we tagged with GFP the coding region of H2A.Z.2 to generate the oligo‐resistant mutants. An ANOVA test detected differences between all categories (*P*‐value 3.48e‐10). We further studied pairwise differences via chi‐squared tests. While proteins harbouring a single mutation were still able to ameliorate the frequency of micronuclei (to be noted that overexpression of H2A.Z.2 is highly toxic to the cells; therefore, we cannot expect a full rescue of the phenotype), the extent of the rescue was still significantly different from the one provided by the WT protein for the S38T mutant (although this latter mutant was expressed at a lower level). Despite all the double mutants were expressed at the same level as the WT, only A14T/S38T was able to rescue the phenotype to a similar extent as the WT form (Figs 1C and EV1F, and Table EV1). This indicates that these few changes in amino acids have indeed a major biological impact, with A127 being one of the essential amino acids when in combination with an additional mutation.

The acetylation status of H2A.Z has been shown to influence its activity (Millar *et al*, 2006; Halley *et al*, 2010; Procida *et al*, 2021). We therefore wanted to test the role of acetylation (or other post‐translational modifications) for the maintenance of genome stability in mammalian cells. H2A.Z.2 also presents two splice variants: H2A.Z.2.1 and H2A.Z.2.2. The latter lacks the utmost C‐terminal tail but retains the extended H2A.Z acidic patch. We first mutated all the lysines that are subjected to post‐translational modifications in H2A.Z.2.1 to non‐acetylable residues (arginines) (GFP:H2A.Z.2) (Fig 1D indicates the mutated residues; Giaimo *et al*, 2019) the GFP:H2A.Z.2.1^KR^ mutant still localised onto the mitotic chromosomes normally (Fig 1E). To really demonstrate that GFP:H2A.Z.2.1^KR^ mutant is indeed incorporated into nucleosomes, we prepared mononucleosomes from HeLa cells transfected with either GFP:H2A.Z.2.1^WT^ or GFP:H2A.Z.2.1^KR^, separated the histones by SDS–PAGE and detected the presence of GFP by immunoblotting (Fig EV1G–I). Collectively, this evidence demonstrates that, despite the number of substitutions, the GFP:H2A.Z.2.1^KR^ mutant is indeed incorporated into the chromatin. However, the KR mutant construct could not efficiently rescue the micronucleation phenotype caused by H2A.Z.2 depletion (Fig 1F, Table EV2).

We then conducted rescue experiments also with the oligo‐resistant mutant GFP:H2A.Z.2.2^WT^ and its respective KR mutant version (the same mutations were used as for the H2A.Z.2.1 apart from K115R and K120R, as these residues are not present in H2A.Z.2.2). Contrary to H2A.Z.2.1, H2A.Z.2.2 is not enriched on chromatin (Fig EV1J). The lack of recruitment onto the chromatin is not due to its failure of binding to the chaperone. In fact, using a LacO‐LacI tethering system that we established in DT40 cells (Vagnarelli *et al*, 2011), we confirmed that the H2A.Z chaperone RFP:LacI:YL1 (part of both the TIP60 and SRCAP complexes) was able to recruit both isoforms to the same extent (Fig EV1L and M); as it could be expected by the behaviour of this variant, GFP:H2A.Z.2.2 was not able to rescue the phenotype (Fig EV1K).

These results suggest that the H2A.Z.2.1 variant is important for genome stability maintenance playing a key role in chromosome segregation and that post‐translational modifications (acetylation and/or methylation or sumoylation) are also required for this function. However, it is also possible that these changes, although they do not affect the incorporation of the variant into chromatin, they could modify other aspects of the biology of this histone variant that ultimately result in a compromised function.

### H2A.Z.2 regulates sister chromatid cohesion

**Figure 2:**
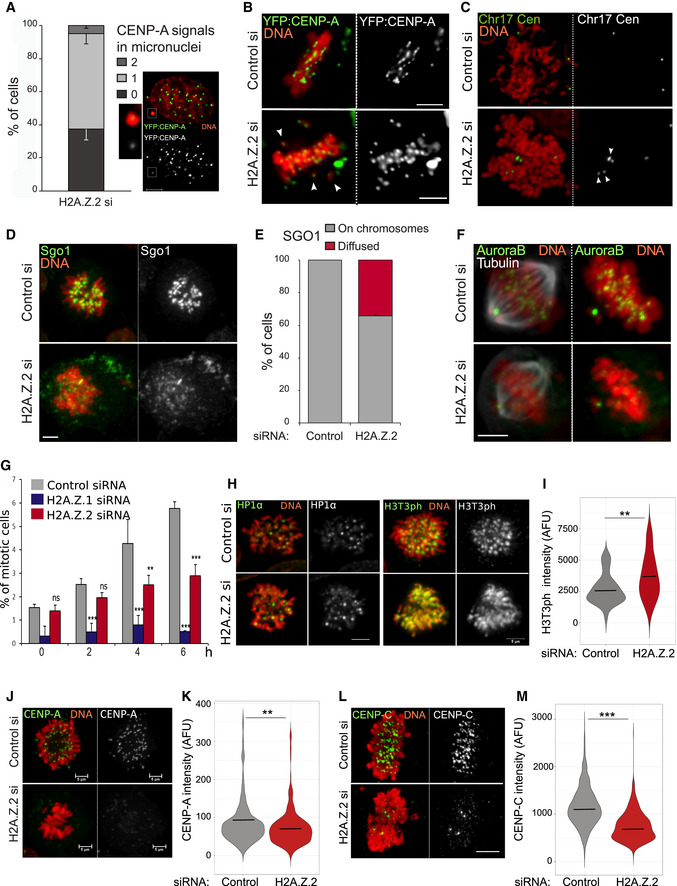
H2A.Z.2 regulates chromosome segregation Quantification of micronuclei containing 0, 1 or 2 CENP‐A signals in HeLa YFP:CENP‐A cells transfected with control and H2A.Z.2 siRNA for 72 h. 160 micronuclei were analysed. Error bars indicate SD of three biological replicates. The image represents an example of a micronucleus containing a single CENP‐A signal. The insets are enlargements of the MN. Scale bar 5 μm.Representative images of metaphases from YFP:CENP‐A (green) HeLa cells treated with control (top) or H2A.Z.2 (bottom) siRNA. The white arrowheads indicate the mis‐aligned chromosomes. Scale bar: 10 μm.Representative images of metaphase spreads from control (top) or H2A.Z.2 (bottom) siRNA‐transfected HeLa cells after FISH with a Chr17 centromeric probe (green). The arrowheads indicate the separated sister chromatids. Scale bar: 10 μm.Representative images of HeLa cells transfected with control (top) or H2A.Z.2 (bottom) siRNA, fixed and stained for Sgo1 (green). Scale bar: 10 μm.Quantification of Sgo1 localisation in prometaphase cells from the experiment in (D). Error bar represents SD of two biological replicates. 35 prometaphase cells were analysed.Representative images of HeLa cells treated as in (D) and stained for Aurora B (green) and alpha tubulin (grey). Scale bar: 10 μm.Mitotic index of HeLa cells transfected with control (grey), H2A.Z.1 (blue) or H2A.Z.2 (red) siRNA and treated with nocodazole for 0, 2, 4 or 6 h. At least 800 cells were analysed for each category. Error bar represents SD of three biological replicates. **P < 0.01; ***P < 0.001, ns, not significant (Chi‐square test).Left panels: Representative images of HeLa cells treated as in (D) and stained for HP1α (green). Scale bar: 10 μm. Right panels: Representative images of HeLa cells treated as in (D) and stained for H3T3ph (green). Scale bar 5 μm.Violin plot of centromeric H3T3ph intensity of prometaphase/metaphase cells from the experiment in (H). The median is shown as bar. Data sets were statistically analysed using the Wilcoxon rank test in R. **P < 0.01.Representative images of HeLa YFP:CENP‐A (green) mitotic cells after control (top) or H2A.Z.2 (bottom) siRNA treatment. Scale bar: 5 μm.Violin plot of centromeric CENP‐A intensity of prometaphase/metaphase cells from the experiments in J. (control si *N* = 197, H2A.Z.2 si *N* = 260). The bar represents the median. Data sets were statistically analysed using the Wilcoxon rank test in R. **P < 0.01.Representative images of HeLa mitotic cells stained for CENP‐C after control (top) or H2A.Z.2 (bottom) siRNA treatment. Scale bar: 5 μm.Violin plot of centromeric CENP‐C intensity of prometaphase/metaphase cells from the experiments in L. (control si *N* = 1,083, H2A.Z.2 si *N* = 686, from 3 biological replicas). The bars represent the median. Data sets were statistically analysed using the Wilcoxon rank test in R. ***P < 0.0001.

**Figure EV2:**
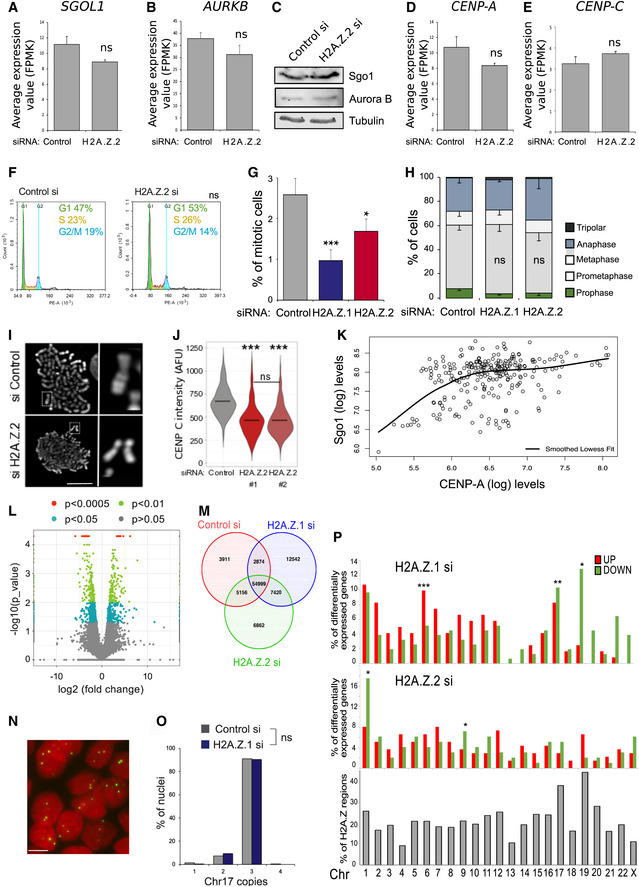
H2A.Z.2 knockdown affects centromeric function *SGOL1* average expression values obtained by RNA sequencing of three biological replicates after control and H2A.Z.2 siRNA treatment. Error bars show the standard deviation (SD). ns, not significant (Student's *t*‐test).
*AURKB* average expression values obtained by RNA sequencing of three biological replicates after control and H2A.Z.2 siRNA treatment. Error bars show the standard deviation (SD). ns, not significant (Student's *t*‐test).Western blot analysis of Sgo1 and Aurora B in mitotic cells after control and H2A.Z.2 siRNA treatment.
*CENP‐A* average expression values obtained by RNA sequencing of three biological replicates after control and H2A.Z.2 siRNA treatment. Error bars show the standard deviation (SD). ns, not significant (Student's *t*‐test).
*CENP‐C* average expression values obtained by RNA sequencing of three biological replicates after control and H2A.Z.2 siRNA treatment. Error bars show the standard deviation (SD). ns, not significant (Student's *t*‐test).Flow cytometry analyses profiles of control and H2A.Z.2 siRNA‐treated HeLa cells. Percentages represent the mean of two biological replicates. Data sets were statistically analysed using Chi‐square test. ns, not significant.Mitotic index of HeLa cells transfected with control, H2A.Z.1 or H2A.Z.2 siRNA. Error bars represent SD of three biological replicates. At least 2,500 cells were analysed for each condition. Data sets were statistically analysed using Chi‐square test. ***P < 0.001; *P < 0.05.Mitotic cells from the experiment in (G) were analysed and classified by mitotic stage. Error bars represent SD of three biological replicates. At least 300 mitotic cells were analysed for each condition. ns, not significant (Chi‐square test).Mitotic spreads of HeLa cells treated with control (top) or H2A.Z.2 (bottom) siRNA. #1. The panel on the right shows a magnification of a chromosome from the box in the left panel (Scale bar 10 μm).Violin plot of centromeric CENP‐C intensity of prometaphase/metaphase cells from HeLa cells treated with control, H2A.Z.2#1 or H2A.Z.2#2 siRNA (control si *N* = 243 H2A.Z.2 si#1 *N* = 80, H2A.Z.2 si#2 *N* = 502). The bars represent the median. Data sets were statistically analysed using the Wilcoxon rank test in R. ***P < 0.0001; ns, not significant.Graph showing the correlation between YFP:CENP‐A signals and the intensity of Sgo1 signals at the centromeres of prometaphase chromosomes after H2A.Z.2 depletion. The black line represents the lowest smoothed fit.Volcano plot representation of differentially expressed genes in the H2A.Z.1‐depleted cells vs the H2A.Z.2‐depleted cells data sets. *Y*‐axis represents the ‐log10 of the *P*‐value. Coloured points mark the genes with significantly increased or decreased expression. *X*‐axis represents the log2 value of the fold change: points with log2 < 0 indicate downregulated genes in the H2A.Z.2‐depleted cells data set compared with the H2A.Z.1‐depleted cells data set; log2 > 0 indicate upregulated genes (Student's *t*‐test).Venn diagram of the regions identified by ATAC‐seq in control si, H2A.Z.1 si‐ and H2A.Z.2 si‐treated HeLa cells.Representative image of HeLa nuclei after FISH with a Chr17 centromeric probe (green) (Scale bar 10 μm).Quantification of the number of FISH signals/nucleus in control (grey) or H2A.Z.1‐depleted (blue) cells. At least 500 nuclei were analysed per condition. Data sets were statistically analysed using Fisher exact test. ns, not significant.Frequency of genes with altered expression (upregulated—red and downregulated—green) per chromosome after H2A.Z.1 (top panel) or H2A.Z.2 (middle panel) depletion. Bottom panel shows the percentage of H2A.Z‐containing regions/chromosome. Data sets were statistically analysed using Fisher exact test. *P < 0.05, **P < 0.01, ***P < 0.001.

We next set out to understand the molecular mechanisms underlying the micronuclei formation in H2A.Z.2‐depleted cells. The presence of micronuclei could be the result of either chromosome mis‐segregation or DNA damage arising from double‐stranded breaks (DSB) and the production of chromosome fragments. To test these hypotheses, we knocked down H2A.Z.2 in a stable cell line that expresses the centromeric histone variant CENP‐A tagged with YFP (YFP:CENP‐A) and analysed the presence of centromeric signals in the micronuclei. As shown in Fig 2A, the majority of micronuclei have at least one CENP‐A signal, indicating that they possibly contain chromosomes rather than DNA fragments. We therefore inspected prometaphases of H2A.Z.2‐depleted cells and noticed that some mis‐aligned chromosomes had two CENP‐A signals—indicating the presence of a whole chromosome—while others presented a single signal—suggesting that they are single chromatids (Fig 2B, white arrows). The latter could suggest that the origin of micronuclei in H2A.Z.2‐depleted cells is caused by a premature sister chromatid separation, rather than by merotelic attachments or error correction defects; this hypothesis was also supported by the analyses of mitotic chromosome spreads (Fig EV2I). In order to corroborate this observation, chromosome spreads of cells depleted for H2A.Z.2 were subjected to fluorescence in situ hybridisation (FISH) with a probe against the centromeric region of chromosome 17. In control cells, only three FISH signals are observed (the HeLa cell line used has three copies of chromosome 17) but, following H2A.Z.2 depletion, the number of FISH signals in mitosis doubled (Fig 2C); this indicates that in H2A.Z.2‐depleted cells sister chromatids are prematurely separated in mitosis.

In mammalian cells, centromeric cohesin removal is prevented until anaphase onset by Shugoshin (Sgo1) (McGuinness *et al*, 2005). In order to investigate whether H2A.Z.2 depletion affected chromosome segregation via altering Sgo1 loading at centromeres, we stained H2A.Z.2‐ and control‐depleted HeLa cells with Sgo1 antibodies. In 40% of prometaphase cells, Sgo1 failed to localise to the centromeres and appeared dispersed (Fig 2D and E). However, protein and mRNA levels of SGOL1 remained unchanged (Fig EV2A and C). In a similar way, Aurora B, that together with INCENP, survivin and borealin from the chromosomal passenger complex (CPC), was mislocalised in prometaphase/metaphase upon H2A.Z.2 depletion (Fig 2F) without any significant mRNA or protein level changes (Fig EV2B and C). Due to these results, we investigated the mitotic progression in H2A.Z.2‐depleted cells, but we only found a slight decrease in the mitotic index and no significant difference in the distribution of mitotic phases (Fig EV2F–H). A compromised spindle assembly checkpoint (SAC) could be compatible with a lack of mitotic block. We therefore tested the ability of cells depleted of H2A.Z.2 to arrest upon treatment with the spindle poison nocodazole. While control siRNA‐treated cells could efficiently arrest with nocodazole and the mitotic index increased over the course of the treatment, H2A.Z.2‐depleted cells could not (Fig 2G); this indicates that the spindle assembly checkpoint is compromised and H2A.Z.2 plays a role in the SAC maintenance. HP1α could represent the link between all these factors. In fact, HP1α and Sgo1 are both involved in recruiting the CPC to the centromere (Kelly & Cowley, 2013; Wang *et al*, 2010; Yamagishi *et al*, 2010; Abe *et al*, 2016; Ruppert *et al*, 2018) and HP1α has been shown to be able to bind H2A.Z‐containing nucleosomes *in vitro* (Whittle *et al*, 2008; Ryan & Tremethick, 2018; Fan *et al*, 2004). To test this hypothesis, we analysed the presence of HP1α at the centromere in mitotic cells after control or H2A.Z.2 depletion. However, at this level of resolution, we could not detect any changes in the centromeric levels of HP1α between the two samples (Fig 2H). The other pathway that contributes to the localisation of the CPC to the centromere is the binding of survivin to the phosphorylated H3T3 (Kelly *et al*, 2010). We therefore analysed the level of H3T3 phosphorylation in control and H2A.Z.2‐depleted mitotic cells. Interestingly, we found a significant increase in the level of H3T3ph in H2A.Z‐depleted chromosomes (Fig 2I) with a localisation that was not only limited to the centromeric region but also more diffused onto the chromosome arms (Fig 2H, right panels). This suggests that H2A.Z.2 depletion leads to defects in the centromeric chromatin. The link between H2A.Z and H3T3 phosphorylation is not known, and therefore, we conclude that H2A.Z.2 represents a novel pathway for the recruitment or maintenance of the CPC and/or Sgo1 to the centromere in mitosis.

Since published studies on the analyses of CENP‐A chromatin have identified an enrichment of the H2A.Z variant in this fraction (Foltz *et al*, 2006), we investigated whether depletion of H2A.Z.2 would affect centromere maintenance. Analyses of CENP‐A levels using the YFP:CENP‐A cell line showed that CENP‐A is reduced at centromeres of H2A.Z.2‐depleted mitotic chromosomes (Fig 2J and K) but CENP‐A mRNA is unchanged (Fig EV2D). This observation could also explain why some micronuclei in the experiment analysed in Fig 2A do not have a kinetochore signal. We therefore asked the question if other kinetochore markers were altered in H2A.Z.2‐depleted chromosomes. We quantified CENP‐C levels at the kinetochores of control and H2A.Z.2‐depleted mitotic chromosomes and observed a significant decrease in CENP‐C upon H2A.Z.2 siRNA (Fig 2L and M) but not a decrease in CENP‐C mRNA (Fig EV2E). This observation was also confirmed using an independent oligo targeting H2A.Z.2 (Fig EV2J). Reduced CENP‐A levels could be at the basis of a dysfunctional kinetochore and lead to the decrease in Sgo1 and Aurora B; alternatively, the two pathways could be independent. To better understand this aspect, we analysed the correlation between Sgo1 and CENP‐A levels in H2A.Z.2‐depleted cells: the data show that a component of the dependency seems to correlate with CENP‐A levels but they also reveal that other factors could be playing a role in the amount of Sgo1, particularly at the higher levels (Fig EV2K). We therefore propose that H2A.Z.2 could affect different pathways that ultimate lead to a compromised kinetochore function.

Altogether, the data suggest that in human cells the H2A.Z.2.1 paralogue, possibly regulated by its post‐translational modification status, is necessary for the maintenance of a functional centromere and that its removal affects the recruitment of several chromosome segregation‐related proteins to the centromere. This therefore represents a novel pathway, and the identification of the molecular details will represent a new direction of investigation.

### H2A.Z.1 and H2A.Z.2 have distinct roles in gene expression regulation

Several studies have linked H2A.Z to transcription regulation (Gevry *et al*, 2007; Rispal *et al*, 2019; Vardabasso *et al*, 2015). We therefore wanted to investigate whether some of the effects we detected in genome stability could be explained by alteration of specific transcriptional pathways upon H2A.Z.2 depletion. In this context, we also wanted to compare the similarities and differences between the two paralogues in respect to gene transcription.

**Figure 3:**
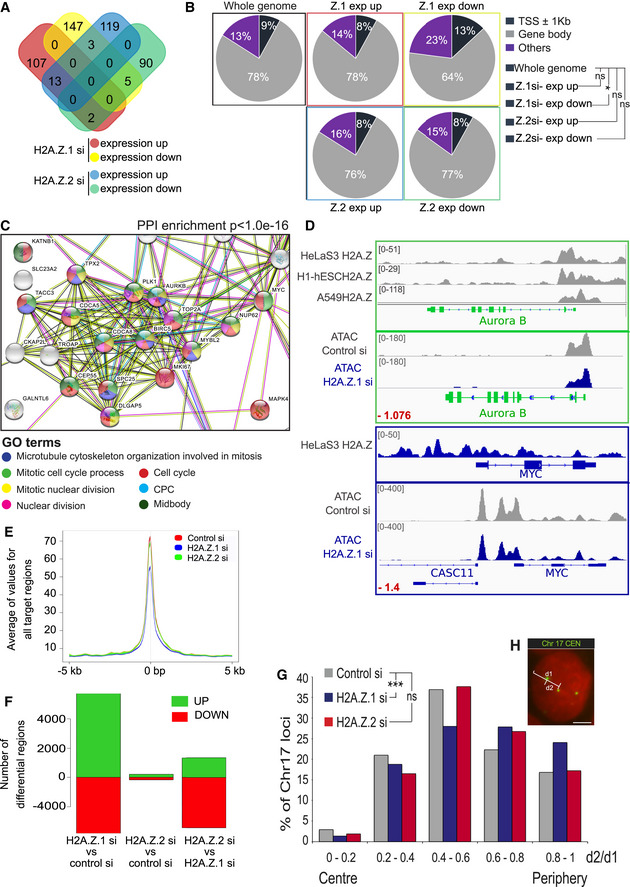
H2A.Z.1 regulates the expression of cell cycle genes Gene expression of three biological replicates of HeLa cells transfected with control, H2A.Z.1 or H2A.Z.2 siRNA was analysed by RNA sequencing. Venn diagram shows the number of significant (*P* < 0.01) differentially expressed genes and the overlap between each set of genes.Pie charts displaying the percentages of H2A.Z peaks at or nearby transcription start sites (TSS) (dark blue), within the gene body (grey) or elsewhere in the whole genome (purple) for the differentially expressed genes following H2A.Z.1 or H2A.Z.2 depletion. A 2‐sample test for equality of proportions was used for the statistical analyses. *P < 0.05; ns, not significant.Downregulated genes following H2A.Z.1 depletion were analysed by STRING. The image shows the cluster of genes with Gene Ontology (GO) terms related to cell cycle. PPI, protein–protein interaction.IGV analyses of H2A.Z localisation (from ENCODE) on Aurora B (green) and cMYC (blue) genes showing H2A.Z enrichment at the TSS and at the upstream region, respectively (bottom panels). IGV analyses of ATAC‐seq peaks for control si and H2A.Z.1 si for Aurora B and MYC genes. The number in red represents the log2Ratio H2AZ.1 si/Control si.Plot representing the average value for all the target regions around the TSS obtained by ATAC‐seq after control, H2A.Z.1 or H2A.Z.2 siRNA.Plot representing the number of differential chromatin accessibility regions in pairwise comparison. Green (up) represents regions with increased accessibility and red (down) with decreased accessibility.Distribution of the position within the nucleus of the centromere of Chr17 from the experiment in (H) calculated as the ratio d2/d1. The ratio between d2 and d1 gives the position of the centromere relative to the centre of the nucleus. The graph represents the percentages of centromeres with distances following within the 5 binning categories. At least 500 nuclei were analysed per condition. Data sets were statistically analysed using Chi‐square test for the distribution of signals among the 5 categories. ***P < 0.001.Representative image of a HeLa nucleus after FISH with Chr17 centromeric probe (green). The distance of the FISH signals from the nucleus periphery was calculated as follows: the distance from the centre of the nucleus to the periphery (d1) and the distance from the centre of the nucleus to the FISH signal (d2) were measured. (scale bar 5 μm).

We conducted RNA‐seq analyses in HeLa cells following H2A.Z.1 or H2A.Z.2 depletion. Our results revealed that no changes in the expression pattern of genes related to chromatin dynamics or cell cycle regulation were present in H2A.Z.2‐depleted cells (Fig EV2A–D), thus strongly indicating that H2A.Z.2 function in chromosome segregation is transcription independent. However, we observed a striking difference in gene expression changes between the two variants. To analyse the features of these genes, we focused on those showing high statistically significant changes in expression (*P* < 0.01) and a fold change > 2. H2A.Z.1 depletion resulted in 147 downregulated genes and 107 upregulated genes; H2A.Z.2 depletion resulted in 90 downregulated genes and 119 upregulated genes. Only 5 and 13 genes were in common between the two variants among the upregulated and downregulated gene lists, respectively (Figs 3A and EV2L). Our results therefore suggest non‐overlapping roles for these two paralogues in gene regulation. To investigate these differences in greater detail, we analysed the distribution of H2A.Z within the genes affected by the two paralogues depletion. Based on published ChIP‐seq data sets (as no antibody is able to discriminate between the two paralogues, ChIP‐seq data sets contain genomic regions occupied by either), we compared the distribution of H2A.Z at three different regions (i—the gene body, ii—at transcription start sites ± 1 kb (TSS) and iii—other parts of the gene) for the whole genome and the four different groups of genes that change expression upon H2A.Z.1 or H2A.Z.2 depletion: upregulated or downregulated genes after H2A.Z.1 or H2A.Z.2 depletion. Interestingly, H2A.Z is enriched at the TSS of genes that are downregulated only after H2A.Z.1 depletion (Fig 3B). This possibly suggests that, in cycling cells, H2A.Z.1 has a more pronounced role in maintaining an open chromatin at active promoters than H2A.Z.2.

In order to assess the changes in chromatin that occur upon H2A.Z.1 and H2A.Z.2 depletion, we conducted ATAC‐seq analyses. These analyses also reveal a significant difference between the effect of the two variants on chromatin: depletion of H2A.Z.1 led to considerable changes in the chromatin status of cells while depletion of H2A.Z.2 only to small variations (Fig 3E and F). Again, the overlaps between the two variants depletion show distinct patterns for each variant (Fig EV2M), thus indicating that they do play distinct roles in chromatin biology. Using STRING analyses, we identified a highly significant protein–protein interaction (PPI) enrichment (*P* = 10^−16^) only for the downregulated genes of H2A.Z.1‐depleted cells. Most of these genes are linked to gene ontologies (GO) terms associated with cell division and mitosis and include MYC, MKI67 and AURKB (Fig 3C). This is in agreement with a decreased mitotic index observed in H2A.Z.1‐depleted cells (Figs 2G and EV2G), suggesting that cells are not cycling. Three out of the four CPC components were significantly downregulated following H2A.Z.1 knockdown (Aurora B, borealin and survivin). Furthermore, analysis of ENCODE/Broad Chip‐Seq data shows that H2A.Z is indeed mainly localised at the promoters of both CPC members and cMYC (Fig 3D).

These results indicate that H2A.Z.1 in HeLa cells is involved in cell cycle progression by promoting the expression of cell cycle‐related genes. Analyses of the ATAC‐seq data upon H2A.Z.1 depletion revealed that indeed changes occurred in the upstream regions of both cMYC and Aurora B compared with the control RNAi (Fig 3D).

Several genes differentially expressed upon H2A.Z.1 knockdown are localised on chromosomes 6 (*P* < 0.001), 17 (*P* < 0.01) and 19 (*P* < 0.05) (Fig EV2P). Interestingly, chromosomes 17 and 19 are the ones with the highest content of H2A.Z bound chromatin (as identified by ChIP) (Fig EV2P—bottom panel); however, no changes in copy number were observed for chromosome 17 (Fig EV2N and O). A quite different distribution was observed for H2A.Z.2. Chromosomes occupy specific territories in the nucleus that are characteristic for each cell type and linked to gene expression (Croft *et al*, 1999; Finlan *et al*, 2008). In order to assess whether chromosome positioning could have been affected by H2A.Z.1 depletion, we analysed the localisation of chromosome 17 using a centromeric FISH probe (Fig 3G and H): high d2/d1 ratios represent signals located at the nuclear periphery, while low d2/d1 ratios represent signals located towards the centre of the nucleus. The results show that following H2A.Z.1 depletion, but not H2A.Z.2, chromosome 17 position is shifted towards the nuclear periphery; this position effect could be linked to the repression of many genes on this chromosome. Alternatively, the major reorganisation of chromatin upon H2A.Z.1 RNAi, as revealed by ATAC‐seq analyses, could in turn affect the nuclear positioning of chromosome territories.

We therefore conclude that H2A.Z.1 and H2A.Z.2 have distinct effects on chromatin organisation, regulate the expression of different subsets of genes and that H2A.Z.1 is specifically involved in cell cycle progression.

### H2A.Z.1 depletion causes G1 arrest and cell senescence

**Figure 4:**
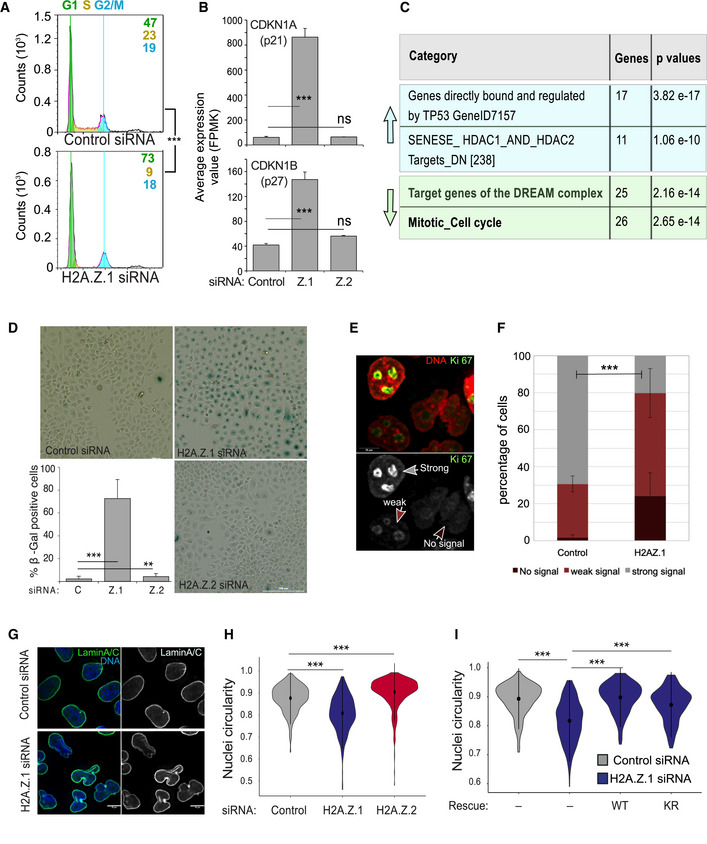
H2A.Z.1 depletion causes G1 arrest and cell senescence Flow cytometry analysis profiles of control (top) and H2A.Z.1 (bottom) siRNA‐treated HeLa cells. Percentages represent the mean of two biological replicates. Data sets were statistically analysed using Chi‐square test. ***P < 0.001.
*CDKN1A* (top) and *CDKN1B* (bottom) average expression values obtained by RNA sequencing of three biological replicates after control, H2A.Z.1 (Z1) or H2A.Z.2 (Z2) siRNA treatment. Error bars show the standard deviation (SD). ***P < 0.001 (Student’s *t*‐test).GSEA analyses of the upregulated (light blue upwards arrow) and downregulated (light green downwards arrow) genes upon H2A.Z.1 siRNA indicating the number of genes for category and the relative *P*‐values.Representative images of HeLa cells transfected with control, H2A.Z.1 or H2A.Z.2 siRNA and stained for senescence‐associated β‐galactosidase (blue). Scale bar 200 μm. At the bottom‐left, quantification of the percentage of β‐galactosidase‐positive cells. At least 500 cells were counted for each experiment. Error bars show the standard deviation (SD) from two biological replicates. **P < 0.01; ***P < 0.001 (Student’s *t*‐test).Representative images of HeLa cell stained for Ki‐67. The arrows indicate the different staining patterns quantified: strong signal (grey arrow), weak signal (light brown arrow) and no signal (dark brown arrow; scale bar 10 μm).Quantification of the experiment in (E). Error bars indicate the standard deviation from three biological replicates. Data sets were statistically analysed using a Chi‐square test. ***P < 0.001.Representative images of HeLa cells transfected with control (top) or H2A.Z.1 (bottom) siRNA and stained for Lamin A/C (green). Scale bar = 10 μm.Violin plots of the nuclear circularity index of HeLa cells transfected with control (grey), H2A.Z.1 (blue) or H2A.Z.2 (red) siRNA. The nuclear circularity was analysed using the NIS Elements AR Analysis software (NIKON). At least 140 nuclei from three biological replicates were analysed for each condition. Mean and SD are shown. Data sets were statistically analysed using the Wilcoxon rank test in R. ***P < 0.001.Violin plots of the nuclear circularity index of HeLa cells transfected with control (grey) or H2A.Z.1 (blue) siRNA either alone or in combination with siRNA‐resistant GFP:H2A.Z.1^WT^ (WT) or GFP:H2A.Z.1^KR^ (KR) plasmids. The nuclear circularity was analysed using the NIS Elements AR Analysis software (NIKON). At least 140 nuclei from three biological replicates were analysed for each condition. Mean and SD are shown. Data sets were statistically analysed using the Wilcoxon rank test in R. ***P < 0.001.

**Figure EV3:**
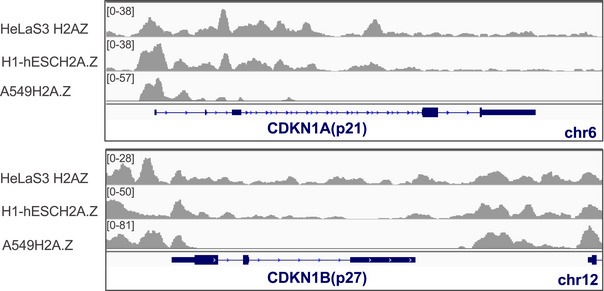
H2A.Z.1 expression levels correlate with neuroblastoma cancer progression IGV analyses of H2A.Z localisation on *CDKN1A* and *CDKN1B* showing H2A.Z enrichment at the TSS.

H2A.Z.1 depletion triggers the downregulation of proliferation‐associated genes such as Ki‐67 and Myc leading to a marked decrease in mitotic index (Figs 2G and EV2G). In fact, FACS analyses of H2AZ.1‐downregulated cells revealed a significant change in the profile with an increase in G1 phase and a reduction in S (Fig 4A), thus suggesting a G1/S arrest. H2A.Z.1‐depleted cells do not proceed through the cell cycle. The pathways involved in the G1 checkpoint are well characterised; therefore, we checked whether the list of genes affected by H2A.Z.1 depletion included some of the known regulators. This was indeed the case: both the cyclin‐dependent kinase inhibitor 1A (CDKN1A) (p21) and 1B (CDKN1B) (p27) were significantly upregulated upon H2A.Z.1 but not H2A.Z.2 RNAi (Fig 4B). Interestingly, both these genes have H2A.Z at their promoters (Fig EV3A). This phenotype was at contrast with the one obtained after H2A.Z.2 depletion where no changes in the cell cycle profile were obtained (Fig EV2F).

To corroborate these findings, we conducted a GSEA analyses of genes that are upregulated and downregulated upon H2A.Z.1 depletion.

These analyses revealed a significant enrichment in genes that are directly bound and regulated by TP53 (*P* = 3.82 e‐17) for the upregulated genes: this again suggests a p53‐mediated cell cycle arrest. On the contrary, among the genes that are downregulated, there was a significant enrichment for mitotic and cell cycle genes and target genes of the DREAM repressive complex that promotes cell cycle exit by repressing cell cycle genes (Uxa *et al*, 2019; *P* = 2.65e‐144 and 2.16 e‐14, respectively, Fig 4C); this further indicates a block in the cell cycle programme upon H2A.Z.1 depletion. As p21 can mediate cellular senescence via p53‐dependent and p53‐independent pathways (Abbas & Dutta, 2009; Qian & Chen, 2010), we investigated whether H2A.Z.1‐depleted cells were in a senescence status by assessing the expression of senescence‐associated β‐galactosidase. We indeed found that 80% of H2A.Z.1‐depleted cells were β‐Gal positive (Fig 4D). As an independent marker for senescence, we also quantified the levels of Ki‐67 in H2A.Z.1‐depleted cells and found a significant increase in cells with weak or no staining for this proliferation marker (Fig 4E and F).

These transcriptional and cell cycle changes were also associated with the presence of an aberrant nuclear shape (Fig 4G), a defect not evident in H2A.Z.2‐depleted cells. To quantify the phenotype, we analysed the nuclear circularity of control, H2A.Z.1‐ and H2A.Z.2‐depleted cells using the NIS Elements AR Analysis software (NIKON); the results showed a significant decrease in the circularity of nuclei upon H2A.Z.1 knockdown (Fig 4H). To confirm that the phenotype was specifically due to H2A.Z.1 depletion and to analyse whether its post‐translational modifications were necessary for this function, we generated GFP‐tagged oligo‐resistant constructs for H2A.Z.1^WT^ and H2A.Z.1^KR^: both constructs were able to rescue the phenotype (Fig 4I). These data indicate that H2A.Z.1 is required to maintain a round nuclear shape, but its post‐translational modifications are dispensable for this function. H2A.Z.2 also alters the roundness of the nuclear shape but this is due to the presence of micronuclei close to the main nucleus rather than by the irregular overall morphology.

### H2A.Z.1 regulates cell cycle progression via MYC

**Figure 5:**
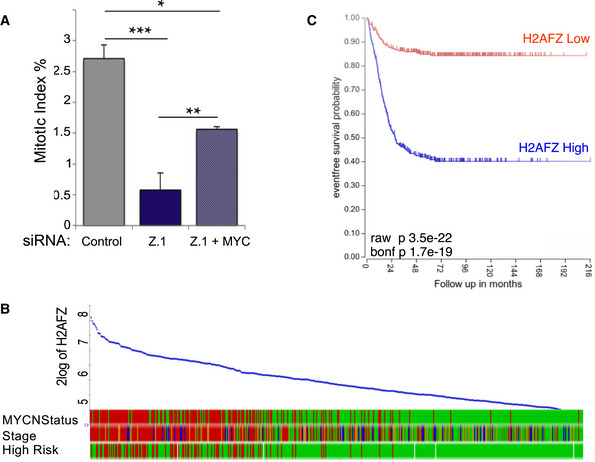
H2A.Z.1 effect on cell cycle is linked to MYC expression Mitotic index (number of mitotic cells/mitotic + interphase cells) of HeLa cells after control si, H2A.Z.1 si or H2A.Z.1 si + GFP:cMYC. Error bars represent standard deviation from two biological replica (control si *N* = 1,033; H2A.Z.1 si *N* = 1,042; H2A.Z.1 si + GFP:cMYC *N* = 1,024). Data sets were statistically analysed using Chi‐square test. *P < 0.05; **P < 0.01; ***P < 0.001.Correlation of H2AFZ (H2A.Z.1) expression levels with MYCN status, cancer stage and risk incidence in neuroblastoma patients obtained from R2 genomics.Kaplan–Meier survival curve for neuroblastoma patients with low (red) or high (blue) expression of *H2AFZ* (H2A.Z.1) obtained from R2 genomics.

Since we have revealed a decrease in cMYC in the RNA‐seq experiments together with a change in the chromatin upstream upon ATAC‐seq analyses, we wanted to test if the cell proliferation block could be due to MYC. We therefore expressed MYC in cells depleted of H2A.Z.1 and evaluated the effect on the cell cycle by analysing the mitotic index. As previously mentioned, H2A.Z.1‐depleted cells have a major decrease in the mitotic index but MYC overexpression can overcome the cell cycle block and significantly increase cell proliferation (Fig 5A).

Because our data indicate a role of H2A.Z.1 in the maintenance of cell proliferation via MYC regulation, we reasoned that these results could have important implications in cancer. MYC activation is the most frequent molecular alteration observed in human cancers, and among those, neuroblastoma represents a classic example. Amplification of the MYCN transcription factor is a common feature of advanced neuroblastoma and associated with a poor outcome of the disease. We therefore investigated whether a correlation between H2A.Z.1 and MYCN exists in neuroblastoma tumours using the R2 genomic platform. We stratified the patients for MYC amplification and stage of the tumour. The results (Fig 5B) clearly show a correlation between H2A.Z.1 level and both MYCN level and clinical stage. Moreover, a Kaplan–Meier analysis also revealed that neuroblastoma patients expressing high level of H2A.Z.1 have a worse prognosis compared with those presenting a low expression level (Fig 5C). This is almost reminiscent of the prognostic value of MYCN itself for this type of cancer.

Therefore, we can conclude that the biological importance of H2A.Z.1 in promoting cell proliferation via favouring Myc transcription and suppression of p21/p27 and could be extremely important in cancer as prognostic indicator but also as possible therapeutical avenue.

**Figure 6:**
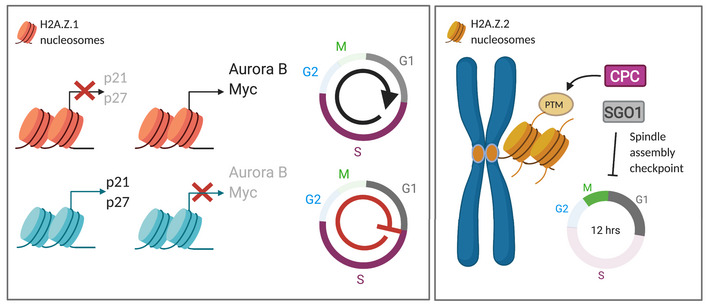
Model for the distinct roles of H2A.Z.1 and H2A.Z.2 in cell cycle regulation (Left panel) H2A.Z.1 promotes the expression of several cell cycle genes whilst repressing the G1/M checkpoint proteins p21 and p27, thus favouring cell cycle progression. Upon downregulation of H2A.Z.1, the expression of these genes is altered leading to G1/S cell cycle arrest and cellular senescence. (Right panel) H2A.Z.2, through its post‐translational modifications, regulates the recruitment of several centromeric proteins and contributes to the regulation of the spindle assembly checkpoint. H2A.Z.2 depletion leads to a defective SAC, premature sister chromatid separation and chromosome segregation errors.

In summary, our data suggest that the two H2A.Z paralogues have distinct roles in the regulation of cell cycle progression: while H2A.Z.1 acts at the G1/S transition by suppressing the expression of p21 and p27 thus facilitating the expression of cell proliferation genes, H2A.Z.2, in a transcription‐independent manner, contributes to the maintenance of centromeric cohesion and recruitment of spindle assembly checkpoint proteins, thus playing a role at the M/G1 transition (Fig 6).

## Discussion

Despite the established importance of the histone variant H2A.Z in many aspects of cell biology and growing evidence suggesting differential roles for the H2A.Z.1 and H2A.Z.2 paralogues in chromatin organisation and function, the majority of the studies thus far have focussed on H2A.Z as a single variant and very little is known about their individual roles (if any) during cell cycle in mammals. In this study, we have shown that H2A.Z.1 and H2A.Z.2 have specific and distinct functions in chromosome segregation and cell cycle progression in human cells.

### H2A.Z.2 is essential for chromosome segregation

H2A.Z.2‐specific depletion in HeLa cells resulted in profound chromosome segregation errors that led to micronuclei formation. Our study has provided clear evidence for the different roles of the splice variants and their post‐translational modifications that are essential for chromosome segregation. The isoform H2A.Z.2 is critical for chromosome segregation, and this function depends on its post‐translational modification status (or it is mediated by other changes linked to the residues that can potentially be modified), since we have demonstrated that both the wild‐type and mutant forms can be incorporated into the chromatin with the same efficiency, but the mutant cannot rescue the segregation defect phenotype. Although the shorter H2A.Z.2.2 variant possesses the acidic patch responsible for its deposition, it lacks the C‐terminal tail, which has been shown to be important in Drosophila (Clarkson *et al*, 1999) and yeast (Wratting *et al*, 2012). The C‐terminal tail contains the A127 residue that seems to be important for chromosome segregation and several residues that can be post‐translational modified and could potentially have an impact either on the stability of the nucleosomes or the recruitment of specific readers. In fact, the mutation of residue 127, although still effective to some extent in ameliorating the micronucleation phenotype, when in combination with either residue 14 or 38 was not. Amino acid 38 is important for the L1 loop structure and nucleosome stability (Horikoshi *et al*, 2013), and the S38T substitution in H2A.Z.1, mimicking T38 found in H2A.Z.2, can rescue the SRCAP‐dependent Floating‐Harbor syndrome (Greenberg *et al*, 2019) defects. The role of the amino acid 14 is still unknown but being an alanine that cannot be phosphorylated (compared to the threonine in the paralogue counterpart), it could suggest that a differential phosphorylation between the two forms may play a critical role, especially during mitosis.

We have investigated the molecular mechanisms behind the chromosome segregation defects. H2A.Z.2‐depleted cells presented impaired loading of CENP‐A, CENP‐C, Sgo1 (protector of centromeric cohesion) and Aurora B (member of the CPC). Interestingly, two H2A.Z‐specific peptides (GDEELDSLIK and ATIAGGGVIPH—note that these sequences are present in both H2A.Z.1 and H2A.Z.2) were found enriched in the CENP‐A nucleosome pull‐downs when compared with those containing histone H3.1 (Foltz *et al*, 2006). This suggests that a specific centromeric CENP‐A containing chromatin could favour the maintenance or deposition of CENP‐A. Interestingly, the majority of H2A.Z deposition at pericentric heterochromatin occurs in G1 (Boyarchuk *et al*, 2014), at the same time as CENP‐A. Treatment with 5’‐aza 2’‐deoxycytidine (5‐Aza) leads to an increased incorporation of both CENP‐A and H2A.Z at pericentric heterochromatin that has been interpreted as a consequence of a disruption in the heterochromatin at the centromere (Boyarchuk *et al*, 2014). However, we do not see a loss of HP1α at the centromere; therefore, our data seem to suggest that H2A.Z.2 incorporation is upstream and can affect CENP‐A deposition or stability. This is a novel aspect that will be further investigated.

We have also observed a decrease in Aurora B levels at centromeres of mitotic chromosomes in cells depleted of H2A.Z.2. It was previously reported that INCENP^360‐876^ could interact *in vitro* with H2A.Z and that the GDEELDSLIKA region was essential for the binding (Rangasamy *et al*, 2003). This could explain the results we have obtained, but it does not account for the specificity; in fact, the interacting region is the same in both paralogues. Our results reveal for the first time a link between H2A.Z.2 and sister chromatid cohesion. The known pathways connecting the CPC and Sgo1 loading are mediated by HP1α (Yamagishi *et al*, 2010; Ruppert *et al*, 2018; Nonaka *et al*, 2002; Inoue *et al*, 2008; Perera & Taylor, 2010; Kang *et al*, 2011). However, H2A.Z.2 depletion does not seem to alter HP1α pericentromeric localisation, thus indicating that we have uncovered a possible novel pathway important for the maintenance of these centromeric proteins in mitosis. The CPC is also maintained at the centromere via the phosphorylation of H3T3 (Kelly *et al*, 2010). Interestingly, depletion of H2A.Z.2 leads to an abnormal distribution of this phosphorylation which spreads into the chromosome arms, while it is normally only localised at the centromeric chromatin. This could contribute to the impaired localisation of the CPC. The link between H2A.Z and the centromeric chromatin structure is not known and could be an important avenue to investigate.

Finally, the data we have provided on the correlation between CENP‐A and the Sgo1 levels at centromere suggest that H2A.Z.2 is a key chromatin player at the intersect of different pathways that altogether contribute to maintain a functional centromere.

### H2A.Z.1 and H2A.Z.2 have separate functions in gene expression regulation

The RNA‐seq analyses also revealed non‐overlapping roles for H2A.Z.1 and H2A.Z.2 in transcription regulation. These distinct effects could be due to the different binding partners reported for H2A.Z.1 and H2A.Z.2: H2A.Z.1 has preference for interacting with BRD2, PHF4, HMG20A and TCF20, whereas H2A.Z.2 has been shown to interact with SIRT1 (Draker *et al*, 2012; Punzeler *et al*, 2017; Lamaa *et al*, 2020). A recent study conducted in U2OS and fibroblast cells analysed the target transcriptomic landscape after individual depletion of each H2A.Z variant; the results showed both distinct and overlapping sets of genes, as well as similar or antagonistic functions, depending on the targets (Lamaa *et al*, 2020). Our data sets differ from the reported one as we obtained an almost non‐overlapping picture of gene expression changes and we observed a significant enrichment of H2A.Z at the TSS of genes that undergo downregulation after H2A.Z.1 depletion. In agreement with this study, our data have shown that H2A.Z.1 depletion resulted in downregulation of a subset of cell cycle‐regulated genes, which explains the cell cycle arrest and low cell division rate we have reported here. However, we did not found downregulation of cell cycle‐related genes after H2A.Z.2 depletion. The ATAC‐seq analyses also revealed distinct effects on chromatin reorganisation upon H2A.Z.1 or H2A.Z.2 RNAi with H2A.Z.1 having a more prominent impact on chromatin than H2A.Z.2.

We have also shown that the cell cycle mis‐regulated genes upon H2A.Z.1 depletion do contain H2A.Z at their promoters, suggesting a direct effect played by the histone variant rather than being a secondary effect; only a few showed changes upon ATAC‐seq analyses. Among those genes, we have identified MYC as one of the key players that mediate H2A.Z.1 effect. In fact, p21, a classic regulator of the G1/S checkpoint, has been reported to be one of the major targets of MYC repression activity (Coller *et al*, 2000). Upon H2A.Z.1 knockdown, MYC expression levels decrease and the ATAC peaks upstream the gene show also a 1.4‐fold decrease; this leads to increased p21 mRNA, activation of the G1/S checkpoint that results in decreased proliferation (low mitotic index), G1 arrest and a senescent phenotype. This effect can be overridden by ectopically expressing cMYC. These data seem to suggest a direct link between MYC and H2A.Z.1

The senescent phenotype is associated with an abnormal nuclear morphology which can be reminiscent of the p53‐mediated cellular senescence pathway mediated by lamin A stabilisation (Yoon *et al*, 2019). However, we did not detect any significant change in lamin A expression level upon H2A.Z.1 RNAi.

Based on the data we have presented on the effect of these paralogues in cell cycle progression and the observed overexpression of H2A.Z in a variety of malignant tumours, including breast (Hua *et al*, 2008), prostate (Slupianek *et al*, 2010), bladder (Kim *et al*, 2013b) cancers and metastatic melanoma (Vardabasso *et al*, 2015), we should consider the relevance of this histone variant in cancer. Here, we have also provided data linking high expression of H2A.Z.1 to MYCN as bad prognostic feature in Neuroblastoma. Moreover, the co‐regulation of MYC, the CPC and its counteracting phosphatase Repo‐Man (CDCA2) by H2A.Z.1 we have here reported, provides further support to the important role of these complexes in cancer where the co‐upregulation of Repo‐Man and Aurora B in tumours is inversely correlated with patient survival (Manzione *et al*, 2020). This again underlines the potential importance of H2A.Z.1 for cancer progression, acting as an oncogene.

Some studies suggested the possibility that it is the balance between the two paralogues that allows for a normal cell homeostasis. In this respect, since we have shown that H2A.Z.2 depletion results in chromosome instability while H2A.Z.1 supports the expression of master cell cycle genes, we can envisage that slight changes in their balance could easily produce an aberrant cancer prone phenotype, on the one hand by supporting proliferation (High H2A.Z.1) and on the other hand by increasing genome instability (Low H2A.Z.2), both beneficial for a tumour cell to evolve. In this respect, it would be interesting to analyse CIN tumours and correlate their evolution with the expression level of H2A.Z.2.

In conclusion, we have demonstrated non‐redundant roles for H2A.Z.1 and H2A.Z.2 in different aspects of cell biology and gene expression, highlighting the importance of studying these variants independently. More studies will be required to unveil the details of the downstream effectors of these paralogues, the chaperones and machineries dedicated to the specific deposition of these variants. This is particularly important for therapeutic purposes, as only targeting the correct variant would offer a proper intervention.

## Materials and Methods

### Cell culture, cloning and transfections

HeLa cells were grown in DMEM supplemented with 10% foetal bovine serum (FBS) and 1% penicillin–streptomycin (Invitrogen Gibco) at 37°C with 5% CO_2_.

DT40 cells carrying a single integration of the LacO array (de Castro *et al*, 2017) were cultured in RPMI1640 supplemented with 10% FBS, 1% chicken serum and 1% penicillin–streptomycin at 39°C and 5% CO_2_.

The HeLa CENP‐A:YFP cell line was kindly provided by Dr Lars E.T. Jansen (Oxford University, UK).

Transient transfections for DT40 in LacO array background were conducted as previously described using GFP‐fused H2A.Z.1, H2A.Z.2.1 and H2A.Z.2.2 (Vagnarelli *et al*, 2011).

For siRNA treatments, HeLa cells were seeded into 6‐well plates, transfected using Polyplus JetPrime^®^ (PEQLAB, Southampton, UK) with the appropriate siRNA oligonucleotides (50 nM) and analysed after 72 h. The siRNAs were obtained from Merck. Control: 5′‐CGUACGCGGAAUACUUCGA‐3′; H2A.Z.1: 5′‐GCCGUAUUCAUCGACACCU‐3′; H2A.Z.2#1: 5′‐AUUUGUAUGUUCUUAGACU‐3′; H2A.Z.2#2: 5′‐GUGACAGUUGUGUGUUGAU‐3′.

For rescue experiments, 1 µg of GFP:H2A.Z.1 DNA or 100 ng of GFP:H2A.Z.2 DNA was used. GFP:H2A.Z.1^WT^, GFP:H2A.Z.2.1^WT^, GFP:H2A.Z.2.2^WT^, GFP:H2A.Z.1^KR^, GFP:H2A.Z.2.1^KR^ and GFP:H2A.Z.2.2^KR^ were synthetised by ProteoGenix (La Haye, France). They were all cloned into pEGFP‐C1 by XhoI/ KpnI, except GFP:H2A.Z.2.1^WT^ and GFP:H2A.Z.2.2^WT^ that were cloned by BglII/ BamHI1 and BglII/ EcoRI, respectively. Single or double mutations were produced with the Q5^®^ Site‐directed Mutagenesis kit (New England Biolabs, UK) using the primers in Table 1.

**Table 1:** List of primer used in the study

Name	Primer	Sequence (5' to 3')
H2A.Z.2.1A14T	Forward	GAAGGCCAAGacaAAGGCAGTATC
	Reverse	CCACTGTCCTTTCCAGCT
H2A.Z.2.1T38S	Forward	ACACTTGAAGtctCGCACCACAAGC
	Reverse	CTGTGGATGCGGCCCACA
H2A.Z.2.1A127V	Forward	GCAGAAAACTgtcTAGGGATCCAC
	Reverse	TGTCCCTTCTTTCCAATC
YL1	Forward	AAGCTTTTATGAGTTTGGCTGGGGGCC
	Reverse	GGATCCTCATTTAATGACAATTTTCTGGCGC

The oligo‐resistant H2A.Z.1 mutants were generated by mutating the following oligo target sequence GGC(G) CGT(R) ATT(I) CAT(H) CGA(R) CAC(H) CTA(L) to GGC(G) AGG(R) ATC(I) CAT(H) AGG(R) CAC(H) CTA(L).

To produce the RFP:LacI:YL1 construct, we first generated a GFP:LacI:YL1 plasmid. The YL1 sequence was obtained by PCR using the primers in Table 1 and cloned into GFP:LacI (de Castro *et al*, 2017) by HindIII/BamHI. GFP was replaced by RFP using NheI/BglII.

GPS‐MYC was a gift from Channing Der (Addgene plasmid # 160130; http://n2t.net/addgene:160130; RRID:Addgene_160130) (Blake *et al*, 2019).

The primers used for the study were acquired from Eurofins Genomics (Germany) and all the restriction enzymes from New England Biolabs (UK).

### Immunofluorescence microscopy

Cells were fixed in 4% PFA and processed as previously described (Vagnarelli *et al*, 2006). Primary and secondary antibodies were used as in Table 2. Fluorescence‐labelled secondary antibodies were applied at 1:200 (Jackson ImmunoResearch). Three‐dimensional data sets were acquired using a wide‐field microscope (NIKON Ti‐E super research Live Cell imaging system) with a numerical aperture (NA) 1.45 Plan Apochromat lens. The data sets were deconvolved with NIS Elements AR analysis software (NIKON). Three‐dimensional data sets were converted to maximum projection using the NIS software, exported as TIFF files and imported into Adobe or Inkscape for final presentation.

**Table 2:** List of antibodies used in the study

Antibody	Source	Cat number	Dilution
Lamin A+Lamin C	Abcam	#ab108595	1:2,500
Alpha‐Tubulin	Sigma	#T5168	1:1,000
Shugoshin	Abcam	#ab58023	1:200
Aurora B/ AIM1	Cell Signalling Technology	#3094	1:200
CENP‐C	Abcam	#ab193666	1:200
Ki‐67	BD Biosciences	#610968	1:100
H3T3ph	Abcam	#ab17352	1:2,000

For the analyses of the mitotic index, cells stained with DAPI and alpha tubulin were used. At least 500 cells per experimental condition for each biological replica were analysed. Chromosome condensation and spindle morphology were used to categorise the different mitotic stages. For analyses of the spindle assembly checkpoint, HeLa cells were transfected with control, H2A.Z.1 or H2A.Z.2 siRNA for 72h and treated with nocodazole (200 ng/ml) for 0, 2, 4 and 6 h.

For quantification of the staining, masks were created around the DAPI nuclei or chromosomes using NIS Elements AR Analysis software. Mean intensity of antibodies signals was extracted and exported to Excel (MS office), and background was subtracted.

Quantification of the kinetochore/centromere staining. (CENP‐A, CENP‐C) 3D stack images were exported and analysed with the Foci quantification plug‐in using Fiji (1_color_auto.ijm) (Ledesma‐Fernandez & Thorpe, 2015). Background was subtracted, and the mean intensity was used to generate the violin plots.

For quantification of enrichment at the LacO locus, four identical circles were designed: one around the LacI spot, two within the nucleus and one outside the cell. Signal intensities were extracted and the mean of the two circles within the nucleus calculated. The outside circle was used as background and subtracted from both the mean nuclear and the LacI spot, and then, the signal intensity from the LacI was normalised relative to the intensity of the nuclear signal.

Violin plots were generated using the ggplot2 package in R.

### Senescence‐associated β‐galactosidase staining

To analyse senescence, HeLa cells were treated with control, H2A.Z.1 or H2A.Z.2 siRNA for 72 h and stained for β‐galactosidase using the senescence β‐galactosidase staining kit (Cell Signalling Technology #9860) following manufacturer’s protocol. Cells were imaged with a BioTek Cytation 5 imaging reader.

### Immunoblotting

Whole cell extracts were prepared by direct lysis in 1× Laemmli sample buffer, separated in SDS–PAGE and transferred onto nitrocellulose membranes. Membranes were blocked with 5% milk in TBS, and primary antibodies were used as following: anti‐GFP (Roche, Cat#11814460001) anti‐H2A.Z (Cell Signalling Technologies, Cat #2781); anti‐HP1α (Millipore, Cat #05‐689) at 2 μg/ml; anti‐Aurora B/AIM1(Cell Signalling Technologies, Cat#3094) at 1:1,000; anti‐Shugoshin (Abcam, Cat #ab58023) at 1:200; anti‐tubulin (Sigma, Cat#T5168); anti‐GAPDH (ProteinTech, Cat#10494‐1‐AP) at 1:10,000. HRP secondary antibodies were used at 1:10,000 and LiCor secondary antibodies (LiCor IRDye 800CW and 680RD) at 1:5,000. Membranes were visualised using either the Bio‐Rad ChemiDoc XRS system or the LiCor Odyssey system.

### FISH

FISH was performed in HeLa cells as previously described (de Castro *et al*, 2017) using a probe against chromosome 17 (Mejia *et al*, 2002).

For the centromere position analyses, a single plane containing each spot was selected. The distance of the spots from the periphery was measured and represented as a fraction of the radius on which each spot belongs.

### Flow cytometry cell cycle analysis

Cells were trypsinised, resuspended and incubated at room temperature for 30 min in 70% ice‐cold ethanol. Cells were centrifuged at 1,000 *g* for 4 min, washed with PBS and the supernatant discarded. The pellet was resuspended in 200 μl of RNase A/PBS (100 μg/ml) and incubated for 2 h at 37°C in the dark. Propidium iodide (Fisher Scientific, P3566) was added at a final concentration of 5μg/ml just before analysing the samples by flow cytometry using the ACEA Novocyte Flow Cytometer. The analysis was performed using the NovoExpress^®^ software.

### Micrococcal nuclease treatment

HeLa cells were transfected with 1 μg of GFP, GFP:H2A.Z.2.1^WT^ or H2A.Z.2.1^KR^ for 24 h. Cells were lysed using lysis buffer (1 M Tris–HCl, 2.5 M NaCl, 10% NP‐40), and the chromatin was extracted by centrifugation at 1,000 *g* for 3 min. Chromatin was flash‐frozen in liquid nitrogen until ready to use. Chromatin was digested with Micrococcal Nuclease (NEB, 37°C, 30 min) in digestion buffer (1 M Tris‐HC, 1 M CaCl_2_, 0.5 M MgCl_2_, 2 M sucrose).

Digested chromatin was resuspended in Laemmli sample buffer and run in a 10% acrylamide gel. Anti‐GFP antibody (Roche, Cat#11814460001) was used to detect the construct and the anti‐H3 C‐terminus (Active Motif, Cat#39052) as a control.

To verify that chromatin was digested, 1% of SDS (final concentration) was added to 50 μl of digested and undigested chromatin. DNA was extracted with phenol chloroform, precipitated with ethanol and analysed in a 1% agarose gel.

### RNA sequencing

RNA was collected from siRNA‐treated HeLa cells and extracted using the RNeasy PowerLyzer Tissue & Cells Kit (Qiagen) according to manufacturer’s protocol. RNA samples were sent to the Wellcome Trust Genomic Centre (Oxford University, Oxford UK) for whole‐genome sequencing, using the Illumina HiSeq4000.

The RNA‐seq data were analysed using open‐source software from the Tuxedo suite, including TopHat2 (Kim *et al*, 2013a) and Cufflinks (Trapnell *et al*, 2010). The paired‐end raw reads were mapped to the human reference genome GRCh38 using the annotations from GENCODE 28 (Harrow *et al*, 2012), with TopHat2 v2.1.1 (Bowtie 2 v2.2.6) under standard conditions. All the sequence and annotation files were downloaded from the Illumina website. The resulting alignments were filtered for high quality hits using SAMtools v0.1.19 (Li *et al*, 2009) with a minimum selection threshold score of 30. Cufflinks v2.2.1 was used to assemble the mapped reads into transcripts and quantify their expression levels. Finally, Cuffdiff, part of the Cufflinks package, was used to identify differentially transcribed genes between samples. Functional enrichment was analysed using String (string‐db.org), while Venn diagrams were performed in the open software FunRich. Volcano plots were performed using the ggplot package in R v3.5.0.

The file containing the H2A.Z ChIP‐seq data used (E117‐H2A.Z.narrowPeak.gz. https://egg2.wustl.edu/roadmap/data/byFileType/peaks/consolidated/narrowPeak/) was filtered using a q‐value threshold of 0.01. The transcription start sites (TSS) are defined by Abugessaisa *et al* (2019).

If two or more H2A.Z regions overlap, they were considered as one region. For each set of genes, the H2A.Z regions that were not within ± 3 kb of any of the genes in that set were excluded, and the remainder were classified into 3 categories: regions that overlap and/or are within ± 3 kb of one or more of the TSS associated with a gene whose expression changes after RNAi knockdown; regions that overlap the loci of genes whose expression changes after RNAi knockdown but not any of the TSS associated with those genes; and regions that do not overlap the TSS nor the loci of genes whose expression changed after RNAi knockdown. The number of H2A.Z regions in each category is divided by the total number of regions for that gene set to give the proportion of H2A.Z regions in each category.

IGV was used for the graphic representation of the ChIP‐seq data on the gene on the gene of interest.

### Differentially expressed gene frequency analyses

To calculate the expected and observed frequency of differentially expressed genes on each chromosome, we used the gene numbers present on each human chromosome and used the chromosome copy number in HeLa cells integrating both the published data sets (Naumova *et al*, 2013) and our own experimental data set for chromosomes 17 (this study) and chromosomes 13 and 14 (de Castro *et al*, 2017).

### **ATAC**‐**seq**

After 72 h treatment with either control, H2A.Z.1 or H2A.Z.2 siRNA, HeLa cells were harvested and frozen in culture media containing FBS and 5% DMSO. Cryopreserved cells were sent to Active Motif to perform the ATAC‐seq assay. The cells were then thawed in a 37°C water bath, pelleted, washed with cold PBS and tagmented as previously described (Buenrostro *et al*, 2013), with some modifications based on (Corces *et al*, 2017). Briefly, cell pellets were resuspended in lysis buffer, pelleted and tagmented using the enzyme and buffer provided in the Nextera Library Prep Kit (Illumina). Tagmented DNA was then purified using the MinElute PCR purification kit (Qiagen), amplified with 10 cycles of PCR and purified using Agencourt AMPure SPRI beads (Beckman Coulter). Resulting material was quantified using the KAPA Library Quantification Kit for Illumina platforms (KAPA Biosystems) and sequenced with PE42 sequencing on the NextSeq 500 sequencer (Illumina).

Reads were aligned using the BWA algorithm (mem mode; default settings) (Li & Durbin, 2009). Duplicate reads were removed; only reads mapping as matched pairs and only uniquely mapped reads (mapping quality ≥ 1) were used for further analysis. Alignments were extended in silico at their 3'‐ends to a length of 200 bp and assigned to 32‐nt bins along the genome. The resulting histograms (genomic “signal maps”) were stored in bigWig files. Peaks were identified using the MACS 2.1.0 algorithm at a cut‐off of *P*‐value 1e‐7, without control file and with the –nomodel option. Peaks that were on the ENCODE blacklist of known false ChIP‐Seq peaks were removed. Signal maps and peak locations were used as input data to Active Motifs proprietary analysis program, which creates Excel tables containing detailed information on sample comparison, peak metrics, peak locations and gene annotations. For differential analysis, reads were counted in all merged peak regions (using Subread), and the replicates for each condition were compared using DESeq2 (Love *et al*, 2014).

The analyses of Neuroblastoma data sets were conducted using the R2: Genomics Analysis and Visualisation Platform (http://r2.amc.nl).

### Statistical analyses

Statistical analyses were performed either in Excel (Chi‐square test) or in R (using the Wilcoxon rank test function, differential expression, lowest smoothing).

## Author contributions

PV and RSG conceptualised the study; IJDC contributed to methodology; RSG, IJDC, HAA, CS, DCK and PV investigated the study; PV and RSG wrote original draft; all the authors wrote review & editing; PV involved in funding acquisition; PV, CS and VV supervised the study.

## Conflict of interest

The authors declare that they have no conflict of interest.

## Supporting information



Expanded View Figures PDF

Table EV1

Table EV2

## Data Availability

All unique/stable reagents generated in this study are available from the Lead Contact with a completed Materials Transfer Agreement. The RNA‐seq data sets and ATAC‐seq generated during this study are available at NCBI http://www.ncbi.nlm.nih.gov/bioproject/629054 and https://www.ncbi.nlm.nih.gov/geo/query/acc.cgi?acc=GSE173113. The microscopy images supporting the current study are available from the corresponding author on request and they will be shared via FIGSHARE.
