# Supplementary material for: Non‐redundant functions of H2A.Z.1 and H2A.Z.2 in chromosome segregation and cell cycle progression
Source: EMBO Rep. 2021 Aug 23;22(11):e52061. doi: 10.15252/embr.202052061 (PMC8567233; doi:10.15252/embr.202052061)
Supplement: Supplementary file 1 — Expanded View Figures PDF [file EMBR-22-e52061-s001.pdf]

Expanded View Figures

Figure EV1. H2A.Z.2.1 knockdown leads to genome instability.

A *H2AFZ* and *H2AFV* average expression values obtained by RNA sequencing of three biological replicates after control, H2A.Z.1 and H2A.Z.2 siRNA treatment. Error bars show the standard deviation (SD). ****P* < 0.001; ns, not significant.

B *H2A* average expression values obtained by RNA sequencing of three biological replicates after control H2A.Z.1 or H2A.Z.2 siRNA treatment. Error bars show the standard deviation (SD). ns, not significant.

C Western blot of HeLa whole cell lysates after control, H2A.Z.1, H2A.Z.2 or H2A.Z.1 + H2A.Z.2 siRNA and probed with anti H2AZ antibody or actin or tubulin. The single depletions were imaged and quantified by LICOR.

D Western blot of HeLa cells transfected with control si + GFP:H2A.Z.1, with control si + GFP:H2A.Z.2, H2A.Z.1 si + GFP:H2A.Z.2 and H2A.Z.2 si + GFP:H2A.Z.1. The blot was probed with H2A.Z (red) and tubulin (green) antibodies and imaged by LICOR. Top panel shows the GFP:H2A.Z and the bottom panel the endogenous H2A.Z.

E Quantification of the number of anaphases with chromatin bridges of HeLa cells after transfection with control, H2A.Z.1 or H2A.Z.2 siRNA. Error bars represent SD of three biological replicates. At least 100 anaphases were analysed for each condition. Data sets were statistically analysed using Chi-square test. **P* < 0.05; ****P* < 0.001.

F Western blot analysis using an anti-GFP, H2A.Z and GAPDH antibodies of HeLa cells transfected with H2A.Z.2 siRNA and the indicated GFP constructs. The blot was imaged by LICOR. The star indicates a non-specific band.

G HeLa cells were transfected with GFP, GFP:H2A.Z.2.1WT (WT) or GFP:H2A.Z.2.1KR (KR), lysed and digested with micrococcal nuclease (MNase) for 30 min to generate mononucleosomes. L: DNA ladder.

H The chromatin fraction (Ch) from (I) was separated on SDS–PAGE, together with the nuclear fraction (NF), and subjected to GFP immunoblotting. Anti-H3 C-terminus antibody was used as a control.

I Western blot analysis using anti-GFP antibody in cells transfected with H2A.Z.2 siRNA and each of the indicated GFP constructs.

J Representative images of prometaphase chromosomes from HeLa cells co-transfected with H2A.Z.2 siRNA and either H2A.Z.2wt or H2A.Z.2KR mutant Scale bar: 5 μm.

K Quantification of the percentage of cells with micronuclei from experiment (J). The error bars represent the SD of three biological replicates (control si *N* = 887; H2A.Z.2 si *N* = 1,005; H2A.Z.2 si + H2A.Z.2.2wt *N* = 465; H2A.Z.2si + KR *N* = 353). Data sets were statistically analysed using Chi-square test: ****P* < 0.001; ns, not significant. Black refers to the comparison with the control RNAi, and blue refers to the comparison with the H2A.Z.2si + H2A.Z.2.2wt data.

L GFP enrichment was calculated as a ratio between the intensity at LacI spot and the mean of two random nuclear spots. Mean and SD are shown. Data sets were statistically analysed using Wilcoxon rank test. ns, not significant.

M Representative images of DT40 cells carrying a LacO array inserted at a single locus co-transfected with RFP:LacI:YL1 (red) and either GFP:H2A.Z.1, GFP:H2A.Z.2.1 or GFP:H2A.Z.2.2 (green). Scale bar: 5 μm.

© 2021 The Authors

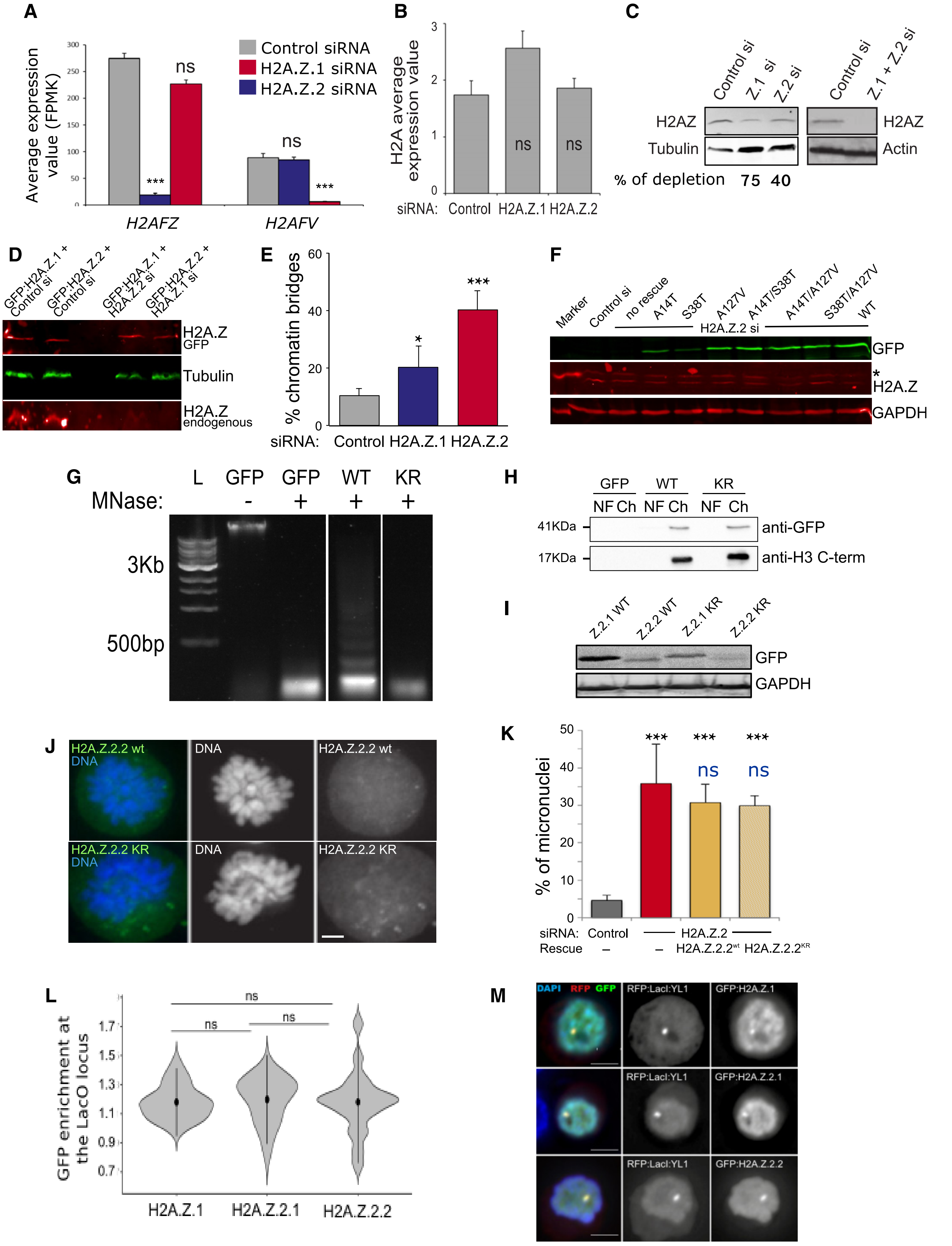

Figure EV1.

 © 2021 The Authors

Figure EV2. H2A.Z.2 knockdown affects centromeric function.

A *SGOL1* average expression values obtained by RNA sequencing of three biological replicates after control and H2A.Z.2 siRNA treatment. Error bars show the standard deviation (SD). ns, not significant (Student's *t*-test).
B *AURKB* average expression values obtained by RNA sequencing of three biological replicates after control and H2A.Z.2 siRNA treatment. Error bars show the standard deviation (SD). ns, not significant (Student's *t*-test).
C Western blot analysis of Sgo1 and Aurora B in mitotic cells after control and H2A.Z.2 siRNA treatment.
D *CENP-A* average expression values obtained by RNA sequencing of three biological replicates after control and H2A.Z.2 siRNA treatment. Error bars show the standard deviation (SD). ns, not significant (Student's *t*-test).
E *CENP-C* average expression values obtained by RNA sequencing of three biological replicates after control and H2A.Z.2 siRNA treatment. Error bars show the standard deviation (SD). ns, not significant (Student's *t*-test).
F Flow cytometry analyses profiles of control and H2A.Z.2 siRNA-treated HeLa cells. Percentages represent the mean of two biological replicates. Data sets were statistically analysed using Chi-square test. ns, not significant.
G Mitotic index of HeLa cells transfected with control, H2A.Z.1 or H2A.Z.2 siRNA. Error bars represent SD of three biological replicates. At least 2,500 cells were analysed for each condition. Data sets were statistically analysed using Chi-square test. ***$P < 0.001$; *$P < 0.05$.
H Mitotic cells from the experiment in (G) were analysed and classified by mitotic stage. Error bars represent SD of three biological replicates. At least 300 mitotic cells were analysed for each condition. ns, not significant (Chi-square test).
I Mitotic spreads of HeLa cells treated with control (top) or H2A.Z.2 (bottom) siRNA. #1. The panel on the right shows a magnification of a chromosome from the box in the left panel (Scale bar 10 μm).
J Violin plot of centromeric CENP-C intensity of prometaphase/metaphase cells from HeLa cells treated with control, H2A.Z.2#1 or H2A.Z.2#2 siRNA (control si $N = 243$ H2A.Z.2 si#1 $N = 80$, H2A.Z.2 si#2 $N = 502$). The bars represent the median. Data sets were statistically analysed using the Wilcoxon rank test in R. ***$P < 0.0001$; ns, not significant.
K Graph showing the correlation between YFP:CENP-A signals and the intensity of Sgo1 signals at the centromeres of prometaphase chromosomes after H2A.Z.2 depletion. The black line represents the lowest smoothed fit.
L Volcano plot representation of differentially expressed genes in the H2A.Z.1-depleted cells vs the H2A.Z.2-depleted cells data sets. *Y*-axis represents the -log10 of the *P*-value. Coloured points mark the genes with significantly increased or decreased expression. *X*-axis represents the log2 value of the fold change: points with log2 < 0 indicate downregulated genes in the H2A.Z.2-depleted cells data set compared with the H2A.Z.1-depleted cells data set; log2 > 0 indicate upregulated genes (Student's *t*-test).
M Venn diagram of the regions identified by ATAC-seq in control si, H2A.Z.1 si- and H2A.Z.2 si-treated HeLa cells.
N Representative image of HeLa nuclei after FISH with a Chr17 centromeric probe (green) (Scale bar 10 μm).
O Quantification of the number of FISH signals/nucleus in control (grey) or H2A.Z.1-depleted (blue) cells. At least 500 nuclei were analysed per condition. Data sets were statistically analysed using Fisher exact test. ns, not significant.
P Frequency of genes with altered expression (upregulated—red and downregulated—green) per chromosome after H2A.Z.1 (top panel) or H2A.Z.2 (middle panel) depletion. Bottom panel shows the percentage of H2A.Z-containing regions/chromosome. Data sets were statistically analysed using Fisher exact test. *$P < 0.05$, **$P < 0.01$, ***$P < 0.001$.

© 2021 The Authors

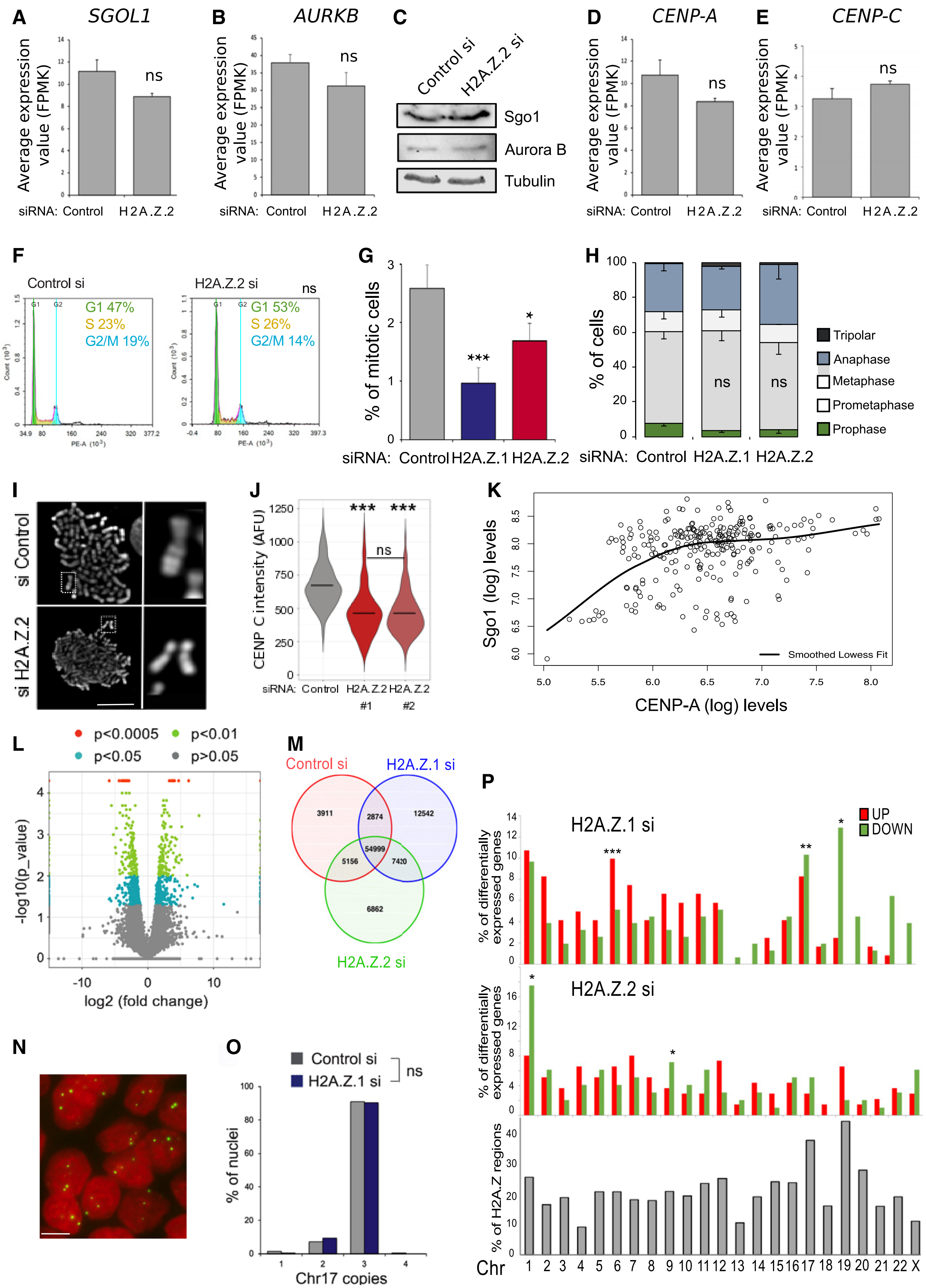

Figure EV2.

 © 2021 The Authors

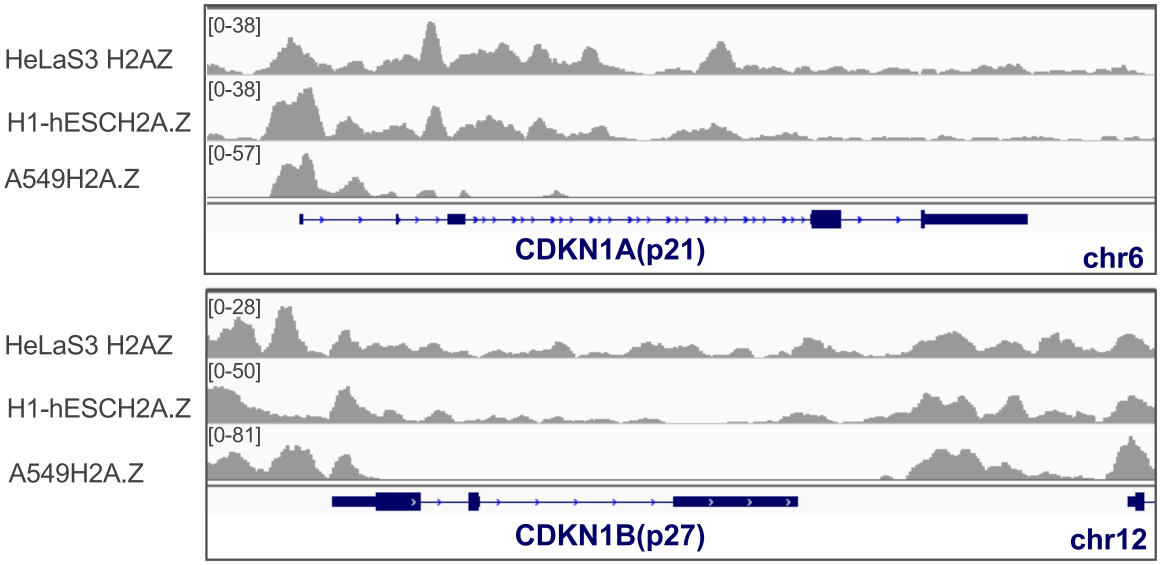

Figure EV3. H2A.Z.1 expression levels correlate with neuroblastoma cancer progression.

IGV analyses of H2A.Z localisation on *CDKN1A* and *CDKN1B* showing H2A.Z enrichment at the TSS.

© 2021 The Authors
