# Supplementary material for: Non‐redundant functions of H2A.Z.1 and H2A.Z.2 in chromosome segregation and cell cycle progression
Source: EMBO Rep. 2021 Aug 23;22(11):e52061. doi: 10.15252/embr.202052061 (PMC8567233; doi:10.15252/embr.202052061)
Supplement: Supplementary file 2 — Table EV1 [file EMBR-22-e52061-s004.docx]

**Table EV1**. Number of cells with (MN) or without (no MN) micronuclei for each biological replicate in figure 1C.

|  |  | Control si | H2A.Z.2 si | H2A.Z.2 si + WT | H2A.Z.2 si + A14T | H2A.Z.2 si + S38T | H2A.Z.2 si + A127V |
| --- | --- | --- | --- | --- | --- | --- | --- |
| Exp1 | **MN** | 10 | 96 | 41 | 72 | 84 | 75 |
|  | **No MN** | 221 | 57 | 112 | 171 | 141 | 154 |
|  | **% MN** | 4.32 | 62.74 | 26.79 | 29.63 | 37.33 | 32.75 |
| Exp2 | **MN** | 4 | 135 | 57 | 63 | 72 | 52 |
|  | **No MN** | 66 | 84 | 102 | 64 | 76 | 105 |
|  | **% MN** | 5.7 | 61.64 | 35.4 | 49.60 | 48.65 | 33.12 |
| Exp3 | **MN** | 18 | 93 | 29 | 81 | 59 | 91 |
|  | **No MN** | 265 | 63 | 164 | 167 | 126 | 172 |
|  | **% MN** | 6.36 | 59.61 | 15.02 | 32.66 | 31.89 | 34.6 |

|  |  | Control si | H2A.Z.2 si | H2A.Z.2 si + WT | H2A.Z.2 si + A14T/S38T | H2A.Z.2 si + A14T/A127V | H2A.Z.2 si + S38T/A127V |
| --- | --- | --- | --- | --- | --- | --- | --- |
| Exp1 | **MN** | 12 | 242 | 172 | 128 | 147 | 166 |
|  | **No MN** | 176 | 25 | 298 | 114 | 190 | 231 |
|  | **% MN** | 6.38 | 51.82 | 36.59 | 52.89 | 43.62 | 41.81 |
| Exp2 | **MN** | 12 | 293 | 178 | 143 | 106 | 111 |
|  | **No MN** | 242 | 396 | 424 | 297 | 117 | 158 |
|  | **% MN** | 4.72 | 42.52 | 29.56 | 32.5 | 47.53 | 41.26 |
| Exp3 | **MN** | 6 | 107 | 45 | 48 | 70 | 113 |
|  | **No MN** | 126 | 100 | 139 | 163 | 161 | 184 |
|  | **% MN** | 4.54 | 51.69 | 24.45 | 22.74 | 30.3 | 38.04 |
| Exp4 | **MN** | 18 | 111 | 71 | 41 | 97 | 50 |
|  | **No MN** | 140 | 75 | 141 | 156 | 150 | 137 |
|  | **% MN** | 11.39 | 59.67 | 33.49 | 20.81 | 39.27 | 26.73 |
