# Supplementary material for: Non‐redundant functions of H2A.Z.1 and H2A.Z.2 in chromosome segregation and cell cycle progression
Source: EMBO Rep. 2021 Aug 23;22(11):e52061. doi: 10.15252/embr.202052061 (PMC8567233; doi:10.15252/embr.202052061)
Supplement: Supplementary file 3 — Table EV2 [file EMBR-22-e52061-s002.docx]

**Table EV2.** Number of cells with (MN) or without (no MN) micronuclei for each biological replicate in figure 1F and EV1 K

|  |  | Control si | H2A.Z.2 si | H2A.Z.2 si + Z.2.1WT | H2A.Z.2 si + Z.2.2WT | H2A.Z.2 si + Z.2.1KR | H2A.Z.2 si + Z.2.2KR |
| --- | --- | --- | --- | --- | --- | --- | --- |
| Exp1 | **MN** | 12 | 128 | 65 | 59 | 60 | 35 |
|  | **No MN** | 214 | 134 | 267 | 118 | 103 | 95 |
|  | **% MN** | 5.3 | 48.85 | 19.57 | 33.33 | 36.80 | 26.92 |
| Exp2 | **MN** | 7 | 138 | 10 | 46 | 46 | 43 |
|  | **No MN** | 240 | 281 | 175 | 90 | 94 | 96 |
|  | **% MN** | 2.8 | 32.93 | 5.4 | 33.82 | 32.85 | 30.93 |
| Exp3 | **MN** | 22 | 94 | 46 | 38 | 40 | 27 |
|  | **No MN** | 392 | 230 | 197 | 114 | 136 | 57 |
|  | **% MN** | 5.3 | 29 | 18.93 | 25 | 22.72 | 32.14 |
