# Supplementary material for: Non‐redundant functions of H2A.Z.1 and H2A.Z.2 in chromosome segregation and cell cycle progression
Source: EMBO Rep. 2021 Aug 23;22(11):e52061. doi: 10.15252/embr.202052061 (PMC8567233; doi:10.15252/embr.202052061)
Supplement: Supplementary file 4 [file EMBR-22-e52061-s003.pdf]

EMBO reports

Non-redundant functions of H2A.Z.1 and H2A.Z.2 in chromosome segregation and cell cycle progression

Raquel Sales Gil, Dorothee Kommmer, Ines de Castro, Hasnat Amin, Veronica Vinciotti, Cristina Sisu, and Paola Vagnarelli
DOI: 10.15252/embr.202052061

Corresponding author(s): Paola Vagnarelli (paola.vagnarelli@brunel.ac.uk)

Review Timeline:

Submission Date:	12th Nov 20
Editorial Decision:	14th Dec 20
Revision Received:	7th Jun 21
Editorial Decision:	23rd Jul 21
Revision Received:	26th Jul 21
Accepted:	3rd Aug 21

Editor: Deniz Senyilmaz Tiebe

Transaction Report:

(Note: With the exception of the correction of typographical or spelling errors that could be a source of ambiguity, letters and reports are not edited. Depending on transfer agreements, referee reports obtained elsewhere may or may not be included in this compilation. Referee reports are anonymous unless the Referee chooses to sign their reports.)

Editorial Decision 14th Dec 2020

Dear Dr. Vagnarelli,

Thank you for the submission of your research manuscript to our journal, which was now seen by three referees, whose reports are copied below.

I apologize for the delay in getting back to you, it took longer than anticipated to receive the referee reports.

We concur with the referees that the proposed non-redundant functions of H2A.Z.1 and H2A.Z.2 are in principle very interesting. However, referees also raise largely overlapping concerns that need to be addressed to consider publication here. In particular,

- The specific effect of H2A.Z.2 on mitosis is not sufficiently supported by the data (referee #2 points 1-3, referee #3 the 1st specific comment).
- The involvement of CENP-A in H2A.Z.2 depletion phenotype is not conclusive (referee #1 point 1, referee #2 point 4).
- The link between H2A.Z.1 and CPC is not sufficiently strong.
- The proposed role of H2A.Z.1 on chromosome 17 localization and chromatin state is not sufficiently supported (referees #2 and #3).
- Quantifications and statistical analyses should be improved (referees #2 and #3)

Should you be able to address all referee concerns, we would like to invite you to submit a revised manuscript. Please address all referee concerns in a complete point-by-point response. Acceptance of the manuscript will depend on a positive outcome of a second round of review. It is EMBO reports policy to allow a single round of revision only and acceptance or rejection of the manuscript will therefore depend on the completeness of your responses included in the next, final version of the manuscript.

We generally allow three months as standard revision time. As a matter of policy, competing manuscripts published during this period will not negatively impact on our assessment of the conceptual advance presented by your study. However, we request that you contact the editor as soon as possible upon publication of any related work, to discuss how to proceed. Should you foresee a problem in meeting this three-month deadline, please let us know in advance and we may be able to grant an extension.

*** Temporary update to EMBO Press scooping protection policy:
We are aware that many laboratories cannot function at full efficiency during the current COVID-19/SARS-CoV-2 pandemic and have therefore extended our 'scooping protection policy' to cover the period required for a full revision to address the experimental issues highlighted in the editorial decision letter. Please contact the scientific editor handling your manuscript to discuss a revision plan should you need additional time, and also if you see a paper with related content published elsewhere.***

IMPORTANT NOTE: we perform an initial quality control of all revised manuscripts before re-review. Your manuscript will FAIL this control and the handling will be DELAYED if the following APPLIES:
1. A data availability section providing access to data deposited in public databases is missing (where applicable).
2. Your manuscript contains statistics and error bars based on n=2. Please use scatter plots in

these cases.

Supplementary/additional data: The Expanded View format, which will be displayed in the main HTML of the paper in a collapsible format, has replaced the Supplementary information. You can submit up to 5 images as Expanded View. Please follow the nomenclature Figure EV1, Figure EV2 etc. The figure legend for these should be included in the main manuscript document file in a section called Expanded View Figure Legends after the main Figure Legends section. Additional Supplementary material should be supplied as a single pdf labeled Appendix. The Appendix includes a table of content on the first page with page numbers, all figures and their legends. Please follow the nomenclature Appendix Figure Sx throughout the text and also label the figures according to this nomenclature. For more details please refer to our guide to authors.

Please note that for all articles published beginning 1 July 2020, the EMBO Reports reference style will change to the Harvard style for all article types. Details and examples are provided at https://www.embopress.org/page/journal/14693178/authorguide#referencesformat

When submitting your revised manuscript, please carefully review the instructions that follow below. Failure to include requested items will delay the evaluation of your revision.

1) a .docx formatted version of the manuscript text (including legends for main figures, EV figures and tables). Please make sure that the changes are highlighted to be clearly visible.

2) individual production quality figure files as .eps, .tif, .jpg (one file per figure).

3) a .docx formatted letter INCLUDING the reviewers' reports and your detailed point-by-point responses to their comments. As part of the EMBO Press transparent editorial process, the point-by-point response is part of the Review Process File (RPF), which will be published alongside your paper. For more details on our Transparent Editorial Process, please visit our website: https://www.embopress.org/page/journal/14693178/authorguide#transparentprocess
You are able to opt out of this by letting the editorial office know (emboreports@embo.org). If you do opt out, the Review Process File link will point to the following statement: "No Review Process File is available with this article, as the authors have chosen not to make the review process public in this case."

4) a complete author checklist, which you can download from our author guidelines (<http://embor.embopress.org/authorguide>). Please insert information in the checklist that is also reflected in the manuscript. The completed author checklist will also be part of the RPF.

5) Please note that all corresponding authors are required to supply an ORCID ID for their name upon submission of a revised manuscript (<https://orcid.org/>). Please find instructions on how to link your ORCID ID to your account in our manuscript tracking system in our Author guidelines (<http://embor.embopress.org/authorguide>).

6) We replaced Supplementary Information with Expanded View (EV) Figures and Tables that are collapsible/expandable online. A maximum of 5 EV Figures can be typeset. EV Figures should be cited as 'Figure EV1, Figure EV2" etc... in the text and their respective legends should be included in the main text after the legends of regular figures.

- For the figures that you do NOT wish to display as Expanded View figures, they should be bundled together with their legends in a single PDF file called *Appendix*, which should start with a

short Table of Content. Appendix figures should be referred to in the main text as: "Appendix Figure S1, Appendix Figure S2" etc. See detailed instructions regarding expanded view here: <http://embor.embopress.org/authorguide#expandedview>.

- Additional Tables/Datasets should be labeled and referred to as Table EV1, Dataset EV1, etc. Legends have to be provided in a separate tab in case of .xls files. Alternatively, the legend can be supplied as a separate text file (README) and zipped together with the Table/Dataset file.

7) We would also encourage you to include the source data for figure panels that show essential data.

Numerical data should be provided as individual .xls or .csv files (including a tab describing the data). For blots or microscopy, uncropped images should be submitted (using a zip archive if multiple images need to be supplied for one panel). Additional information on source data and instruction on how to label the files are available <http://embor.embopress.org/authorguide#sourcedata>.

8) Our journal encourages inclusion of *data citations in the reference list* to directly cite datasets that were re-used and obtained from public databases. Data citations in the article text are distinct from normal bibliographical citations and should directly link to the database records from which the data can be accessed. In the main text, data citations are formatted as follows: "Data ref: Smith et al, 2001" or "Data ref: NCBI Sequence Read Archive PRJNA342805, 2017". In the Reference list, data citations must be labeled with "[DATASET]". A data reference must provide the database name, accession number/identifiers and a resolvable link to the landing page from which the data can be accessed at the end of the reference. Further instructions are available at <http://embor.embopress.org/authorguide#datacitation>.

9) Please make sure to include a Data Availability Section before submitting your revision - if it is not applicable, make a statement that no data were deposited in a public database. Primary datasets (and computer code, where appropriate) produced in this study need to be deposited in an appropriate public database (see <http://embor.embopress.org/authorguide#dataavailability>).

Please remember to provide a reviewer password if the datasets are not yet public.

The accession numbers and database should be listed in a formal "Data Availability " section (placed after Materials & Method) that follows the model below. Please note that the Data Availability Section is restricted to new primary data that are part of this study.

Data availability

The datasets (and computer code) produced in this study are available in the following databases:

- RNA-Seq data: Gene Expression Omnibus GSE46843 (https://www.ncbi.nlm.nih.gov/geo/query/acc.cgi?acc=GSE46843)
- [data type]: [name of the resource] [accession number/identifier/doi] ([URL or identifiers.org/DATABASE:ACCESSION])

*** Note - All links should resolve to a page where the data can be accessed. ***

10) Regarding data quantification, please ensure to specify the name of the statistical test used to generate error bars and P values, the number (n) of independent experiments underlying each data point (not replicate measures of one sample), and the test used to calculate p-values in each figure legend. Discussion of statistical methodology can be reported in the materials and methods section, but figure legends should contain a basic description of n, P and the test applied.
Please note that error bars and statistical comparisons may only be applied to data obtained from at least three independent biological replicates.
Please also include scale bars in all microscopy images.

We would also welcome the submission of cover suggestions, or motifs to be used by our Graphics Illustrator in designing a cover.

I look forward to seeing a revised version of your manuscript when it is ready. Please let me know if you have questions or comments regarding the revision.

Yours sincerely,

Deniz Senyilmaz Tiebe

Deniz Senyilmaz Tiebe, PhD
Editor
EMBO Reports

Referee #1:

In this manuscript by Salas Gil et al, the authors have investigated the distinct roles of H2A.Z.1 and H2A.Z.2 in cell cycle regulation and they claim to have discovered non-redundant functions for each isoform in cell division. Their findings
show that H2A.Z.1 regulates the expression of cell cycle genes
and its depletion leads to a G1 arrest and cellular senescence. On the other hand, H2A.Z.2 appears essential for centromere integrity
and sister chromatid cohesion regulation.

This is an interesting manuscript that shows a novel role for H2A.Z.2, which is independent on transcriptional regulation. I do however have a few issues that need to be addressed: 1) The effects documented in Figure 1 and 2 appear somewhat modest, the authors claim significant effects of the mutants yet I fail to be convinced; 2) The papers cited for the importance of acetylation are only mentioned for yeast work, this need to be updates to what has been done in human cells. Also with the K to R mutants, it is unclear to me which residues have been mutated exactly? This should be discussed properly. In that same line of thought the authors claim that post-translational modifications are essential for genome-stability etc. This need to be revised as the mutations performed could affect a number of functions unrelated to post-tranlational modifications...

Referee #2:

The histone H2A.Z variant has been associated with several cellular functions and is known to occupy the +1 position at many promoters. There are 2 paralogs of H2A.Z that differ by 3 amino acids, H2A.Z.1 and H2A.Z.2. The latter is 4-fold more highly expressed than the former. The individual contributions of the paralogs to the gamut of functions that H2A serves has not been delineated. The current manuscript uses siRNAs selective for the two paralogs to determine if there are different functions for these highly related H2A variants. The authors show that suppression of H2A.Z.2 leads to mitotic defects that they propose is due to reduction in CENP-A at centromere and leads to alteration in SGO1 recruitment and precocious sister chromatid separation. The manuscript also compares the transcriptional functions of the H2A.Z paralogs by RNA-seq and find that the two paralogs regulate largely distinct and non-overlapping sets of genes. Although, this reviewer is not convinced that the effects on mitosis is specific to H2A.Z.2 because of the difference in expression levels between H2A.Z.2 and H2A.Z.1. Finally, the work suggests that the programs controlled by H2A.Z.1 have a unique effect on regulating genes involved in cellular senescence. Overall, the quality of the experiments is high. There are three very interesting observations in the manuscript that merit further study. However, in several cases the work fails to go into sufficient depth to fully substantiate the hypotheses put forward. Specific examples are listed below. The paper may benefit from focusing on either the contribution of H2A.Z.2 to mitosis or H2A.Z.1 and H2A.Z.2 on transcription. Delineating the functional differences between H2A.Z.1 and H2A.Z.2 is an exciting goal, and the manuscript has a good start on addressing this question. Addressing the points below will hopefully provide sufficient mechanistic insight to support publication.

Major points:
1. The ability of exogenously expressed WT H2AFZ or H2AFV to rescue siRNA suppression in the micronuclei assay was very limited and somewhat concerning. This also makes the contributions of the main acid swaps (which is a very good experiment!) very difficult to interpret. The statistical tests in this case are not correctly applied, as comparisons are only made to the un-rescued condition. ANOVA analysis should be applied. This analysis may not find any difference between WT and any of the mutants which would not support the hypothesis.
2. I remain unconvinced that the effects on mitosis shown for suppression of H2A.Z.2 are unique to H2A.Z.2. They may result from suppressing a larger pool of H2.A.Z, since H2A.Z.2 appears to account for 80% of H2A.Z at least at the RNA level. More direct evidence for a selective role of H2A.Z.2 is required to substantiate this hypothesis. Selective ChIP of H2A.Z.2 to centromeres for example (using tagged H2A.Z.2 and H2.A.Z.1). Western blot should be include showing relative level of expression between expressed H2A.Z and endogenous H2A.Z in figure 1C,D.
3. Relevant to the point listed above, percent H2A.Z loss should be assessed at the protein level. Even though the two paralogs cannot be distinguished, the degree of protein loss should be determined, H2AFZ would be predicted to lead to ~80% of H2AZ, and H2AFV, ~20% based on the RNA. Since both siRNAs are extremely efficient at the RNA level, this worth determining. Since it could affect the interpretation of the results.
4. Figure 2 I and J, the image shown in J seems to be an extreme example of CENP-A loss, while most appears to be mild (maybe less than 10-20%) Missegregation events associated with CENP-A loss in HeLa require significant loss of CENP-A (Black et al 2007), so I am not sure that the small CENP-A loss is the reason for SGO1 changes and PSCS.
5. H2A.Z.1 alters the CPC levels, but does this result in changes in CPC function, reduced H3 phosphorylation? There is no clear connection between this observation and the phenotypes observed.
6. ATAC seq should be done determine how the chromatin changes at specific sites that are affected in response to H2A.Z.1 versus H2A.Z.S loss.
7. The idea that H2A.Z.1 alters chromosome 17 localization in the nucleus is an interesting

hypothesis but lacks sufficient analysis in the current manuscript to support it. This could be secondary to a misregulation of genes required for its localization. It would also require that the occupancy of 17 by H2A.Z.1 be different on Chromosome 17 relative to chromosomes not affected, which is not tested in the manuscript.
8. Swap experiments similar to figure 2C should be conducted for the effects of H2A.Z.1 and 2 on transcriptional regulation. The WT and mutants could also be used for ChIP to determine what changes in RNA seq are direct effects.
9. Figure 4 should include effects of H2A.Z.2 on p27 and p21 expression in figure 4B. Additional senescence markers should be used to demonstrate pathway regulation at the protein level. GSEA analysis of changes in senescence pathways should be included from the RNA-seq data in figure 3 to substantiate the process.
10. Knockdown of p27 and P27 in combination with H2A.Z.1 siRNA should be conducted to show that H2A.Z.1 effect on repressing these genes is key to the senescence phenotype.

Minor Issues:
1. The loss of localization of H2A.Z.2.2 may be due to disruption of the histone fold. It would help to include the locations of the histone fold and alpha helixes in the diagram 1E. However, it's not clear that this is relevant to the rest of the paper, and could probably be removed.
2. The authors state that there were not changes in expression of cell cycle or chromatin dynamics genes. It would be good to show the changes in the specific genes examined in figure 2.
3. To assess the degree of sister chromatid separation the authors should show chromosome spreads in control and H2A.Z.2 siRNA treated cells.

Referee #3:

Summary & main findings:

This study investigates the functions of the two histone variant paralogs H2A.Z.1 and H2A.Z.2. Previous knock out studies in chicken DT-40 cells, mouse and yeast indicated differential and non-overlapping functions of H2A.Z.1 and H2A.Z.2. However, information for distinct functions in human cells is currently lacking. To investigate this, the authors perform siRNA knockdown of H2A.Z.1 and H2A.Z.2 in human HeLa cells. Firstly, the authors demonstrate a role for H2A.Z.2 in chromosome segregation. Secondly, the authors show that H2A.Z.1 functions in cell cycle progression. The authors also show that H2A.Z.1 and H2A.Z.2 regulate the expression of different sets of genes, presumably due to the incorporation of each variant at promoters of specific genes. Finally, given its function in cell cycle progression, the authors implicate the H2A.Z.1 expression level as a prognostic marker in cancer. This is an interesting study which provides insight into the different functions two histone paralogs in human cells, which differ by only three amino acids. In general, the data is well presented and technically sound. I suggest the following amendments and/or clarifications before publication.

Specific comments:

- Given that antibodies which can distinguish between H2A.Z.1 and H2A.Z.2 do not exist, the authors confirm the specificity of H2A.Z.1 and H2A.Z.2 depletion using RNA sequencing. However,

the authors could use the GFP-tagged transgenes to confirm specificity of protein depletion. This is an important result as it affects the interpretation of all experiments.

- In Figure 1, the frequency of micronuclei observed in H2A.Z.2 knockdown experiments seems to be quiet variable. In Figure 1B, this number is 20%, while in Figure 1C it is 60% and Figure 1G it is 30%. Similarly, the extent of rescue by WT construct seems variable. The authors should clarify these observations and provide exact numbers.

- In Figure 2A, it would be nice to show representative images of micronuclei containing one CENP-A focus or two CENP-A foci, given that a loss of sister chromatid cohesion is the major proposed mechanism. Can the authors explain what accounts for the almost 40% of nuclei that do not contain a CENP-A focus?

- In Figure 2C, five signals for Chr17 Cen FISH are shown, yet the authors comment in the text that this number doubled. Please clarify. Is it possible that additional foci are due to aneuploidy rather than a loss of sister chromatid cohesion? Also, in Figure 2F, there appears to be a misaligned chromosome in the control siRNA, which is not expected. This image should be replaced if it is not representative.

- The dramatic loss is CENP-A after 72hrs treatment with H2A.Z.2 siRNA is striking. Do the authors know if other centromere or kinetochore proteins localize correctly e.g. CENP-B or -C or if the centromeres/kinetochores make correct microtubule attachments? This would be interesting to examine given that these cells have a compromised SAC and may shed light on how chromosome segregation defects arise at anaphase.

- In Figure 3F, the authors should indicate the statistical significance for each d2/d1 measurement. As presented, it is difficult to appreciate how H2A.Z.1 values are significant, but H2A.Z.2 are not.

- The defects in nuclear circularity observed in the H2A.Z.1 RNAi are striking. From the quantitation present in Figure 4E, it appears that H2A.Z.2 counts are also significantly different from the control, but this is not mentioned in the text. Please explain. Finally, it would be informative if the authors could speculate in the discussion on why such nuclear defects might occur. For example, does H2A.Z.1 affect Lamin expression?

- My final comment relates to potential overlapping functions of H2AZ.1 and H2A.Z.2. Given the demonstrated importance for H2A.Z.2 in genome instability, it is surprising that the authors do not investigate any correlation between H2A.Z.2 and cancer progression/prognosis. Also, on a related note, can the authors expand on why they believe high - as opposed to low, and thus comparable to the siRNA experiments - H2AFZ expression leads to worse outcomes/survival probability for cancer patients? Also surprising is the finding that although H2AZ.1 specifically affects the expression of Aurora B, Borealin and Survivin, it is the H2A.Z.2 knockdown which displays a chromosome segregation phenotype. However, Figure 1 does show that micronuclei were observed in the H2AZ.1 RNAi, possibly indicating chromosome segregation defects. To address potential overlapping functions with H2A.Z.2, the authors should show whether Aurora B, Borealin and Survivin are correctly localised in the H2AZ.1 RNAi.

- Please correct the following typos:
Page 7, second paragraph - splice variants
Page 8, paragraph title should read 'sister chromatid cohesion'
Figure legend 4B should read H2A.Z.1 not H2A.Z.2

We thank all the referees for their time in evaluating the manuscript and the constructive comments. Based on the suggestions, we were able to address their points and prepare a much stronger version of the paper.
Below is our detailed point-to point response (in black) to each of the queries (in blue) and the specification of how we have changed the manuscript accordingly (in green).

Response to Reviewer 1

We thank the referee for the time spent in evaluating the manuscript and finding it and "interesting" manuscript that shows a novel role for H2A.Z.2, which is independent on transcriptional regulation."

The referee expressed some concerns that we have addresses in this revised version.

1_The effects documented in Figures 1 and 2 appear somewhat modest, the authors claim significant effects of the mutants, yet I fail to be convinced.
For the effect of Figure 1, we have carefully looked into the data and realised that the experiments presented in Figure 1 B (of the previous version) had been conducted in a cell line overexpressing GFP:HP1. We have therefore repeated the experiments in a normal HeLa Kyoto cell line. In these new sets of experiments, we have a higher frequency of micronuclei for H2A.Z.2 si. We have also added another independent oligo against the H2A.Z.2 isoform and, although slightly less efficient in the depletion, it shows a similar phenotype. This other oligo has also been used to quantify some of the centromere markers. These new data should address the concerns of the specificity of the effect on chromosome segregation obtained by depletion of H2A.Z.2 (new Figure 1 B).
The rescue experiments have been re-analysed statistically and made the comparison suggested. These analyses still reveal a difference between the different constructs.
The rescues need to be also interpreted in light of the fact that overexpression of H2A.Z.2 is highly toxic for cells (see Figure 1 below), therefore we cannot expect the complete (100%) rescue of the phenotype. We have added a discussion on this point within the text and the text reads as follows "(to be noted that overexpression of H2A.Z.2 is highly toxic to the cells therefore we cannot expect a full rescue of the phenotype)"

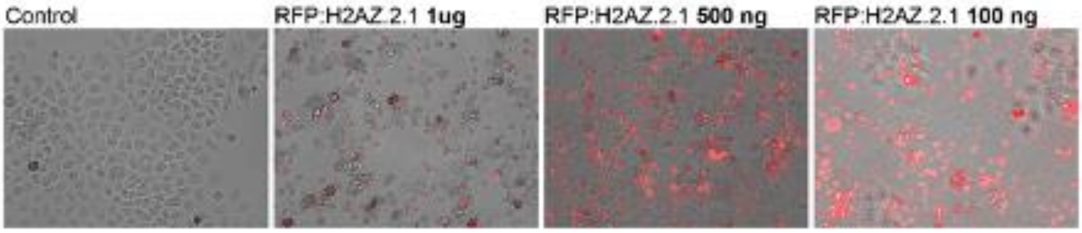

For Figure 2 we are not sure at which of the experiments the referee refers to. The effects on cohesion and the passenger localisation are quite substantial. If the referee refers to the decrease in CENP-A level (although referee #3 considers that "The dramatic loss is CENP-A after 72hrs treatment with H2A.Z.2 siRNA is striking"),

we need to bear in mind that CENP-A is halved at each cell division and there is no turnover aside from the new incorporated protein in G1. In this sense, as it is an RNAi experiment, we need to wait sufficient time to have a depletion of H2A.Z.2 below a threshold that shows an effect and then this will trigger the decrease in CENP-A incorporation. In this respect, it is not surprising that the decrease in CENP-A is not huge, and this small variation is actually expected. However, since referee # 3 suggested also to investigate other kinetochore proteins, we analysed the level of CENP-C. I hope that the referee agrees that the effect on CENP-C is quite substantial (Figure 2 L, M and EV1 J).

2_The papers cited for the importance of acetylation are only mentioned for yeast work, this need to be updates to what has been done in human cells.
We have updated the citations including (Procida, Friedrich et al., 2021).

Also with the K to R mutants, it is unclear to me which residues have been mutated exactly? This should be discussed properly.
We have now referred more explicitly within the text to the scheme in Figure 1E. The text reads as follows "(Figure 1 D indicates the mutated residues)"

In that same line of thought the authors claim that post-translational modifications are essential for genome-stability etc. This need to be revised as the mutations performed could affect a number of functions unrelated to post-translational modification.
We have discussed this point more carefully in the discussion session. The text reads as follows "However, it is also possible that these changes, although they do not affect the incorporation of the variant into chromatin, they could modify other aspects of the biology of this histone variant that ultimately results in a compromised function"

Response to Reviewer 2

We thank the referee for the evaluation of the manuscript and finding that "the quality of the experiments is high" and that "there are very interesting observations". We are also grateful for the suggestions provided that we believe have greatly improved the quality of the manuscript and strengthen the message.

Here below is how we have addressed the referee's points:

1. The ability of exogenously expressed WT H2AFZ or H2AFV to rescue siRNA suppression in the micronuclei assay was very limited and somewhat concerning. This also makes the contributions of the main acid swaps (which is a very good experiment!) very difficult to interpret.
The rescues need to be interpreted in light of the fact that overexpression of H2A.Z.2 is highly toxic for cells (see Figure 1 in response to Referee 1), therefore we cannot expect a complete rescue. However, all the differences are statistically significant and reproducible.
To further support the micronuclei phenotype generated by H2A.Z.2si we have added another independent oligo and showed a significant increase of micronuclei also in this case (new Figure 1 B). This latter oligos shows also the same phenotype in decreasing CENP-C at the kinetochores (new Figure EV2 J) and therefore clearly demonstrates that depletion of H2A.Z.2 compromises the centromere/kinetochore structure.

The statistical tests in this case are not correctly applied, as comparisons are only made to the un-rescued condition.
We have conducted ANOVA analyses and found differences among the samples. We have therefore compared the rescue experiments with each other. The only single rescue mutant that is significantly different from the wt is the S38T, however this latter expresses less in cells, therefore the possible explanation is that it is the level rather than the mutation that effects the results. Among the double mutants, whilst there is a statistically significant difference between the wt and A14T+A127C mutants and also between the wt and the S38T+A127C mutant, there is no statistically significant difference between the wt and the A14T+S38T mutant. This leads us to conclude that this latter mutant is able to act as the wt in the context of the micronuclei phenotype. The significancy of the analyses are reported on the graphs in Figure 1C.

2. I remain unconvinced that the effects on mitosis shown for suppression of H2A.Z.2 are unique to H2A.Z.2. They may result from suppressing a larger pool of H2.A.Z, since H2A.Z.2 appears to account for 80% of H2A.Z at least at the RNA level.
Here the referee is incorrect, it is actually the opposite. As it can be seen by the RNA seq data, the mRNA level of H2A.Z.2 (80 FPMK) is much less than the H2A.Z.1 (270 FPMK) one (EV1A). This is also shown by blot (EV1 C, F). The suppression of H2A.Z.2 leads to a much smaller reduction (as expected) of the total H2A.Z pool compared to the depletion of H2A.Z.1 but still has a very specific phenotype on the centromeric chromatin as shown by using 2 independent oligos and different centromeric and kinetochore markers (Figure 2 H-M and EV1 J).

Western blot should be included showing relative level of expression between expressed H2A.Z and endogenous H2A.Z in figure 1C,D.
We have included the blot showing the endogenous H2A.Z levels in EV1 F.

3. Relevant to the point listed above, percent H2A.Z loss should be assessed at the protein level. Even though the two paralogs cannot be distinguished, the degree of protein loss should be determined, H2AFZ would be predicted to lead to ~80% of H2AZ, and H2AFV, ~20% based on the RNA. Since both siRNAs are extremely efficient at the RNA level, this worth determining. Since it could affect the interpretation of the results.
As suggested by the referee, we have added a blot with the single depletions in Figure EV1 C and quantified the reduction by LICOR. Again, here it shows that the predominant form of H2A.Z in HeLa cells is the H2A.Z.1 as predicted by the mRNA level and that depletion of H2A.Z.2 only reduce a small pool of the total H2A.Z.

4. Figure 2 I and J, the image shown in J seems to be an extreme example of CENP-A loss, while most appears to be mild (maybe less than 10-20%) Mis segregation events associated with CENP-A loss in HeLa require significant loss of CENP-A (Black et al 2007), so I am not sure that the small CENP-A loss is the reason for SGO1 changes and PSCS.
We agree that the role of H2A.Z.2 in centromere maintenance is not limited to CENP-A loading. The correlation analyses we have conducted show some dependency but not complete dependency (this graph has been moved to Figure EV2 K in order to accommodate other analyses conducted for Figure 2). We do believe that the lack of H2A.Z.2 affects two independent pathways that ultimately contributes to the centromere function: one helps CENP-A maintenance and the other the targeting of SGO1/CPC as the correlation analyses shows. In the revised manuscript we have also analysed the level of CENP-C (Figures 2 L,M and EV2 J) and found that is much decreased upon H2A.Z.2 depletion. This further supports a compromised

centromere/kinetochore upon H2A.Z.2 RNAi (also confirmed by another oligos against H2A.Z.2).

5. H2A.Z.1 alters the CPC levels, but does this result in changes in CPC function, reduced H3 phosphorylation? There is no clear connection between this observation and the phenotypes observed.
Maybe here we failed to explain this correctly and we have revised the text accordingly to make this point clearer for the readers.
Depletion of H2A.Z.1 blocks cells in G1 (Figure EV2 F) and therefore the depleted cells do not reach mitosis (as shown by the mitotic index in H2A.Z.1si in Figure 2 G and EV2 G). In this depletion there is a block of the cell cycle and of a cell proliferation programme. MYC levels are downregulated, Ki67 levels are downregulated (now also shown as quantification of the staining by immunofluorescence in Figure EV3 B,C). Myc and Aurora B are coregulated as it is CDCA2 (we have unpublished data on the regulation of CDCA2 by MYC by binding directly to its E box at the promoter). Therefore, this phenotype is consistent with a block of a proliferation programme (see also the GSCA analyses suggested by the referee now added to Figure 4 D).
Aurora B is degraded after mitosis (doi: 10.1128/MCB.25.12.4977-4992.2005) and needs to be replenished before the following division but the H2A.Z.1 depleted cells do not progress beyond G1 therefore the H3T3 phosphorylation, which is mitotic specific, cannot be assessed.

6. ATAC seq should be done determine how the chromatin changes at specific sites that are affected in response to H2A.Z.1 versus H2A.Z.2 loss.
We thank the referee for this suggestion, and we have conducted these analyses. The ATAC data are now shown in Figure 3 D,E,F and again reinforce the differential effect of the two paralogues at chromatin level where H2A.Z.1 is the major component.

7. The idea that H2A.Z.1 alters chromosome 17 localization in the nucleus is an interesting hypothesis but lacks sufficient analysis in the current manuscript to support it. This could be secondary to a misregulation of genes required for its localization.
It would also require that the occupancy of 17 by H2A.Z.1 be different on Chromosome 17 relative to chromosomes not affected, which is not tested in the manuscript.
We do not claim that the re-localisation of chromosome 17 is physically mediated by H2A.Z.1. We do provide the evidence that depletion of H2A.Z.1 (but not of H2A.Z.2) alters chromosome 17 position within the nucleus and that chromosome 17 contains several genes that are repressed upon H2A.Z.1 RNAi. In this respect, these analyses do support the conclusion. This observation also fits well with the current knowledge that upon repression some genes or chromosomes move toward the nuclear periphery. In addition, as the ATAC seq revealed that there are significant changes in chromatin accessibility upon H2A.Z.1 depletion compared to H2A.Z.2, these can also account for the changes in chromosome positioning observed.
We have now separated the genes that are upregulated form the ones that are downregulated in the final table and compared the distributions to the occupancy of H2A.Z.1 on the different chromosomes as suggested. We did find some correlations with Chr17 and Chr19 being the ones with the highest H2A.Z occupancy and also the ones with a significative representation of genes that change expression upon H2A.Z.1 RNAi. . However, we do not feel we can really put much emphasis on these correlations as the ChIP seq data cannot distinguish between the H2A.Z.1 and H2A.Z.2, therefore we cannot draw compelling conclusions at this point.

8. Swap experiments similar to figure 2C should be conducted for the effects of H2A.Z.1 and 2 on transcriptional regulation.
We thank the referee for this suggestion. We agree that it would be interesting to unveil, and we have attempted to answer the question in different ways but, unfortunately, we could not get a conclusive answer that we feel comfortable to add to the paper. We explain below the reason:
We tried to use as read out the level of p21 (Figure below Panel A)[Figures for referees not shown.]. We can clearly see that depletion of H2A.Z.1 caused a p21 increase and that the transfection of the wt decreases the intensity of p21. Aside from this, we could not obtain reproducible and constant responses for the mutants. This might be caused by the level of the transgene expression and/or the level of the depletion. It seems also that the GFP constructs gets cleaved, therefore it is not easy to discriminate the level of protein between depletion and rescue.
We also tried to look at another parameter such as beta gal and quantify by LICOR the experiment but, again we obtained inconsistent results (one example is given in Figure 1 B).

The difference between this isoform compared to the H2A.Z.2 is the expression level. This is the most prominent form (>80% of H2A.Z) and, possibly, to rescue the function a significant level of expression must be reached. In a rescue experiment this can vary quite considerably, thus making the interpretation of the results very difficult.
We believe that, in order to perform this experiment correctly, we will need to invest a considerable amount of time in preparing an endogenously degron-tag cell line and then express the constructs in an inducible manner form the same locus.; only way meaningful comparisons can be made. These tools will require 1 year to be prepared and we think that we have added already a lot of information to this paper to send a clear message on the different role of the two H2A.Z variants.

9. Figure 4 should include effects of H2A.Z.2 on p27 and p21 expression in figure 4B.
We have included the data in Figure 4B.

- Additional senescence markers should be used to demonstrate pathway regulation at the protein level.
We have added the staining and quantification for Ki67 (Figure EV3 B,C)

- GSEA analysis of changes in senescence pathways should be included from the RNA-seq data in figure 3 to substantiate the process.
We thank the referee for the suggestion. We have added the analyses to Figure 4 D that clearly shows enrichment for TP53 target genes for the genes upregulated upon

H2A.Z.1si and enrichment for cell cycle genes and target of the DREAM complex for genes that are downregulated. This is really in line with the phenotype observed.

10. Knockdown of p27 and P27 in combination with H2A.Z.1 siRNA should be conducted to show that H2A.Z.1 effect on repressing these genes is key to the senescence phenotype.
Actually, upon ATAC seq we have identified that p27 and p21 did not change chromatin accessibility, however MYC did in a similar way as its mRNA. MYC has already been linked to the repression of p21, therefore we tested if re-introducing MYC by overexpression could override the cell cycle block. Indeed, this was the case and let to a significant increase in mitotic index even in a H2A.Z.1 RNAi background.

Minor Issues:

2. The authors state that there were not changes in expression of cell cycle or chromatin dynamics genes. It would be good to show the changes in the specific genes examined in figure 2.
The data are presented in Figure EV2 A-D.

3. To assess the degree of sister chromatid separation the authors should show chromosome spreads in control and H2A.Z.2 siRNA treated cells.
Figure 2 C represents chromosome spreads and FISH allows to clearly identify the degree of separation at a specific locus. However, we have added an extra image of spreads in Figure EV1 G.

Referee #3:

We thank the referee for evaluating out manuscript and finding it “an interesting study which provides insight into the different functions two histone paralogs in human cells” and also recognising that “ the data is well presented and technically sound”.
We are also grateful for the suggestions made that we believe have contributed to make a stronger manuscript.

1_ Given that antibodies which can distinguish between H2A.Z.1 and H2A.Z.2 do not exist, the authors confirm the specificity of H2A.Z.1 and H2A.Z.2 depletion using RNA sequencing. However, the authors could use the GFP-tagged transgenes to confirm specificity of protein depletion. This is an important result as it affects the interpretation of all experiments.
We have conducted the suggested experiments and added the blots in Figure EV1D. This is not surprising as the mRNA results suggested that the levels were not affected by the others. These proteins are coded by two different genes. Although they evolved by duplication, the sequences have diverged. The oligo against H2A.Z.1 has been chosen in a region which contains several changes in the sequence that will render the oligo not effective (see Figure below – red box). The oligos against H2A.A.2 are in the 3’UTR which are totally divergent.

Range 1: 1 to 866 Graphics

▼ Next Match ▲ Previous Match

NW Score	Identities	Gaps	Strand
-3797	713/3030(24%)	2168/3030(71%)	Plus/Plus

```
Query  1    ACGGGGT--ATTGTCCGGCTCCGGCGGCGGCGGTCGGTGCTGCGAGAGCGGCGGCGG--C  56
Sbjct  1    GCAGTTTGAATCG--CGG-TGCGACGAAGGAG-TAGGTGGTGGGATCTCACCGTGGGTCC  56

Query  57   GGCGCGGGTCGGCAGCGGGAGGGCGCGCGGCCGAGCGGAGGCGGAGTCGGCGCCGAGAAC  116
Sbjct  57   GATTAGCCTTTTCT-CTGCCTTGCTTGCTT--GAGCTTCAGCGGAATT-----CGAAA--  106

Query  117  ATGGCTGGAGGCAAAGCTGGAAAGGACAGTGGGAAGGCCAAGGCTAAGGCAGTATCTCGC  176
Sbjct  107  -TGGCTGGCGGTAAGGCTGGAAAGGACTCCGGAAAGGCCAAGACAAAGGCGGTTTCCCGC  165

Query  177  TCACAGAGAGCTGGGCTACAGTTTCCTGTGGGCCGCATCCACAGACACTTGAAGACTCGC  236
Sbjct  166  TCGCAGAGAGCCGGCTTGCAGTTCCCAGTGGGCCGTATTCATCGACACCTAAAATCTAGG  225

Query  237  ACCACAAGCCATGGAAGGGTGGGTGCCACTGCTGCCGTGTACAGTGCTGCGATTCTGGAG  296
Sbjct  226  ACGACCAGTCATGGACGTGTGGGCGCGACTGCCGCTGTGTACAGCGCAGCCATCCTGGAG  285

Query  297  TACCTCACTGCAGAGGTGCTGGAGCTGGCAGGTAATGCTTCTAAGGATCTCAAAGTAAAG  356
Sbjct  286  TACCTCACCGCAGAGGTACTTGAACTGGCAGGAAATGCATCAAAAGACTTAAAGGTAAAG  345

Query  357  CGTATCACTCCGCGTCACTTGCAGCTTGCAATCCGTGGTGATGAAGAGTTGGATTCTCTT  416
Sbjct  346  CGTATTACCCCTCGTCACTTGCAACTTGCTATTCGTGGAGATGAAGAATTGGATTCTCTC  405

Query  417  ATCAAGGCTACCATAGCTGGGGGTGGTGTGATCCCTCACATCCACAAATCTCTGATTGGA  476
Sbjct  406  ATCAAGGCTACAATTGCTGGTGGTGGTGTCATTCCACACATCCACAAATCTCTGATTGGG  465

Query  477  AAGAAGGGACAGCAGAAAACTGCTTAGAGGGATGCTTTAACCAACCCTCTTCCTCCCCGT  536
Sbjct  466  AAGAAAGGACAACAGAAGACTGTCTAAAGG-ATGCCTGGA---------TTCCT------  509

Query  537  CATTGTACTGTAACTGGGACAGAAGAAATAATGGGGATATGTGGAATTTTTAACAACAGT  596
Sbjct  510  ---TGT----TATCT----CAG-------------------------------------  520
```

H2AZ.1

GCAGTTTGAATCGCGGTGCGACGAAGGAGTAGGTGGTGGGATCTCACCGTGGGTCCGATTAGCCT
TTTCTCTGCCTTGCTTGCTTGAGCTTCAGCGGAATTCGAA**ATGGCTGGCGGTAAGGCTGGAAAGGACTCCG**
GAAAGGCCAAGACAAAGGCGGTTTCCCGCTCGCAGAGAGCCGGCTTGCAGTTCCCAGTGGGCCGTAT
TCATCGACACCTAAAATCTAGGACGACCAGTCATGGACGTGTGGGCGCGACTGCCGCTGTGTACAGCG
CAGCCATCCTGGAGTACCTCACCGCAGAGGTACTTGAACTGGCAGGAAATGCATCAAAAGACTTAAAGG
TAAAGCGTATTACCCCTCGTCACTTGCAACTTGCTATTCGTGGAGATGAAGAATTGGATTCTCTCATCAAGGCT
ACAATTGCTGGTGGTGGTGTCATTCCACACATCCACAAATCTCTGATTGGGAAGAAAGGACAACAGAAGACT
GTCTAAAGGATGCCTGGATTCCTTGTTATCTCAGGACTCTAAATACTCTAACAGCTGTCCAGTGTTGGTGATTC
CAGTGGACTGTATCTCTGTGAAAAACACAATTTTGCCTTTTTGTAATTCTATTTGAGCAAGTTGGAAGTTTAATT
AGCTTTCCAACCAACCAAATTTCTGCATTCGAGTCTTAACCATATTTAAGTGTTACTGTGGCTTCAAAGAAGCTATT
GATTCTGAAGTAGTGGGTTTTGATTGAGTTGACTGTTTTTAAAAAACTGTTTGGATTTTAATTGTGATGCAGAAGT
TATAGTAACAAACATTTGGTTTTGTACAGACATTATTTCCACTCTGGTGGATAAGTTCAATAAAGGTCATATCCCAAA

H2AZ.2

ACGGGGTATTGTCCGGCTCCGGCGGCGGCGGTCGGTGCTGCGAGAGCGGCGGCGGCGGCGCGGGTCG
GCAGCGGGAGGGCGCGCGGCCGAGCGGAGGCGGAGTCGGCGCCGAGAAC**ATGGCTGGAGGCAAAGCTG**
GAAAGGACAGTGGGAAGGCCAAGGCTAAGGCAGTATCTCGCTCACAGAGAGCTGGGCTACAGTTTCCTGT
GGGCCGCATCCACAGACACTTGAAGACTCGCACCACAAGCCATGGAAGGGTGGGTGCCACTGCTGCCGTG
TACAGTGCTGCGATTCTGGAGTACCTCACTGCAGAGGTGCTGGAGCTGGCAGGTAATGCTTCTAAGGATCT
CAAAGTAAAGCGTATCACTCCGCGTCACTTGCAGCTTGCAATCCGTGGTGATGAAGAGTTGGATTCTCTTAT
CAAGGCTACCATAGCTGGGGGTGGTGTGATCCCTCACATCCACAAATCTCTGATTGGAAAGAAGGGACAG
CAGAAAACTGCTTAGAGGGATGCTTTAACCAACCCTCTTCCTCCCCGTCATTGTACTGTAACTGGGACAGAA
GAAATAATGGGGATATGTGGAATTTTTAACAACAGTTAAATGGAAAAGCATAGACAATTACTGTAGACATGA
TAAAAGAAAC**ATTTGTATGTTCTTAGACT**CGAAGTTTGATAAAAGTACCTTTTCATGTG**GTGACAGTTGTGTGTT**
GATTGGCTAGGTTTCTCCCGTGTGTTTTATACAAAAATGGAATTGATAAACCATTTTTTACAAAATTAATTTGTCT
CAAAACTGTTCTGTTCATGATGTATTAGAAATATTTTACTCAGACTTTAAATATTTTAAATCTCAGATTGGTTATTCA
GAGTAACCTTAGAACAGAAATTGGGAATATATCTTTACAATGATTGATACCATGGTATATTGACTCTTAGATGCTATT
GATCTGTAGCACCATTTTTTACAAACGACTAAGGAAAAAACCTGCCAATTAAATCATGATATGCCATCAATTATGA
GACATCCCAATTTGAGAGATGTTAGATTATAGAAAAGTATGCATTTATGACTGAAATGGTAGTGGAATTATTTGAAT
TCTACACCAAGCACTTACCATGTGCCAGGCCCTTTGCAGAGTGCTCTACTGACCAAGAAAGTTGTTGCTGCCA
CATTATAGATGTGGAGCCTAAGGGTCACAGAAATTGTGTGCTATGCCAAAAAACATTGAACTGGTAGATAGAAAATG
ACAGAGCTAGGATTCAAACCTAGATCTGGCTGACTCCAGAGCCTAGTTTTACCTGGAATTGATGTTCAGTTTAT
CAAAGGTTTCTCCTTTTGGTTTAAAATCCCAATTTTTGGCCTGGCATTGTGGTTTACGCCTGTAATCCCAACACTT
CGGGAGACCGAGGCTGGTGGAACACTTGAGGTCAGGAGTTTGAGACCAGCCTGGCCAACATGGTAAAACGC
CGTCTCGGCCAGGCGCGGTGGCTCACGCCTGTAATCCCAGCACTTTGGGAGGCCAAGGTGGGTGAATCAC
GAGGTCAGGAAATCGAGACCATCCTGGCTAACATGGTGAAACCCCGTCTCTATTTAAAAAAATACAAAAAATTAGC
CGGGTGTGGTGGCACGCGCCTGTAGTCCCAGCTACTCAGGAGGCTGAGGCATGAGAATGACGTGAACCCGG
GAGGCGGAGCTTGCAGTGAGCCAAGATGGCGCCACTGCACTCCAGCTTGGCGACTGAGCAAGACTCCCTCT
CAAAACAAACAAAAAAAAGTCTCTACTAAAAATACAGAAATTAGCCAGGCATGGTACACACATGTTGTCCCAAC
TACTTGGGGCACTGGGGCACAAAAAATCACTTGAACCCAGGAGGCAGAGGTTGCAGTGAGCCAAGATCACGC
CACTACACTCCAGCCTAGGTGACAGAGTGTGACTCTGTCTCAAAAAAAAAAAATCCCAACTTTTAGTAGTCTCT
TAGTCATGCAATAACAGTAATTTGTACAATCTTTTAAAAATTATATTTATTTATCAGTTTCTAAGAAACTTTTTTGT
TTGTTTTGAGACAGGCTCTTGCTCTTTTGCCCAGGTTGAAGTGCAGTGGCATGATCCTGGCTCACTGCAGCCTC
CACCTCTCAGGCCCAAGCAATCCTCTTACCTCAGCCCTGCAAATAGCTGGGACCACAGGCACATGCCACCAT
ACCTGGCTAATTTTTTTTATTTATGTAAGAGACAGAGGTCTCCCTATGTTGCCCAGGTTGGTATTGAACTCCTGGCT
CAAGCCATCCTCCCACCTTGGCCTCCCAAAGTACTGGGATTATAGGCATAAGCCACCATGCCCTGCGCTAAGT
AACTGTTACTTGAGTTAATGTACTAGTTAATTGACCCTTAGAAAATTATATTTTTCTGCTTGCAAGTCTTCATTAAA
GAAGGAAATTTTAAAATATTTTATAGTATAATGCTATCCAAACTCATTTTTAAAAACATTTTATTATGGAAATTTTCA
CAAATGCACAAAAAGAATAGCAGAATGAAGCTCTGTGTACCCATCCTCCAACAGCTGTCCTGTGGTCAGTCTTGT
TTACCTGCATCCCCACCTATCCCCTGCCCCAACCCACAGGGATCAGTTTGAGTCCCATTAACAGGCATAGTAT
TTTCATGTCTGTGTGATCAGAGACATTCAAATATAACTCCAAAGATAGGGTACTTTTTTGAACATAACCACAATAC
CATTGTGTAAGACTGCTAAAACATTTTTTGATGCCAAGTACCAGTCAATATTCAAACTTCCTGATTGTCTCGTAA
GTTTTTTTTAACAGTTGGTTTATTCGAGTCAAGATCCAGGCAAGATCTAGATCTTGCATTTTGTTAATATAATCTATA
GATTTAACTTTCCTGTTTTTAATTTTTGAAGAAACTAAGTTGTTTGTCCTATAGAATTGTCCTTCAGTGGATTTTACT
GAATGTATCCTAATGGGATCATGTACACCTTTTCTGTCCCCTATATGTTCTATAAACTGACAGATCTAGAGGG

ORF H2A.Z.2_1 siRNA
H2A.Z.1 siRNA H2A.Z.2_2 siRNA

2_In Figure 1, the frequency of micronuclei observed in H2A.Z.2 knockdown experiments seems to be quiet variable. In Figure 1B, this number is 20%, while in Figure 1C it is 60% and Figure 1G it is 30%. Similarly, the extent of rescue by WT construct seems variable. The authors should clarify these observations and provide exact numbers.

We thank the referee for this point, and we do agree that in Figure 1 B the levels were quite low. We have looked into these experiments that were conducted at the very beginning of this story and realised that for that set of experiments we used a HeLa cell line that overexpresses HP1α. This fact could be interesting per se and we might explore in the future. We have therefore repeated the experiments in a normal HeLa kyoto cell line and found values similar the ones in experiment 1C. We have

also included another oligo. However, it is possible that the efficacy of the transfection could vary form experiments, thus contributing to the variability observed. We have added the data in a supplementary table.

3- In Figure 2A, it would be nice to show representative images of micronuclei containing one CENP-A focus or two CENP-A foci,
We have added an image of micronuclei as panel in Figure 1A.

4_ given that a loss of sister chromatid cohesion is the major proposed mechanism. Can the authors explain what accounts for the almost 40% of nuclei that do not contain a CENP-A focus?
We have added an explanation in the text: the text reads as follows: “This observation could also explain why some micronuclei in the experiment analysed in Figure 2 A do not have a kinetochore signal.”

5_ Also, in Figure 2F, there appears to be a misaligned chromosome in the control siRNA, which is not expected. This image should be replaced if it is not representative.
Actually, we have chosen this image on purpose because this is a normal step during chromosome alignment and, in normal cells, these mis-aligned chromosomes have more Aurora B.

6_The dramatic loss is CENP-A after 72hrs treatment with H2A.Z.2 siRNA is striking. Do the authors know if other centromere or kinetochore proteins localize correctly e.g. CENP-B or -C or if the centromeres/kinetochores make correct microtubule attachments?
This would be interesting to examine given that these cells have a compromised SAC and may shed light on how chromosome segregation defects arise at anaphase.
We thank the referee for this comment, and we have conducted some experiment to check the localisation and the level of CENP-C at the kinetochores. Our new data presented in Figures 2 K,L and EV1 G clearly show a strong decrease in CENP-C at the kinetochores, thus really demonstrating that H2A.Z.2 depletion compromises the centromeres/kinetochores organisation. Moreover, we have also investigated H3T3ph and found an abnormal distribution of this marker that could account for the mis-localisation of the CPC.

7_ In Figure 3F, the authors should indicate the statistical significance for each d2/d1 measurement. As presented, it is difficult to appreciate how H2A.Z.1 values are significant, but H2A.Z.2 are not.
We have explained the analyses in the legend of the figure.

8_Finally, it would be informative if the authors could speculate in the discussion on why such nuclear defects might occur. For example, does H2A.Z.1 affect Lamin expression?
We have a section in the discussion about this aspect and, following the referee’s comment, we have now added a clarification about Lamin A expression. The paragraph reads as follow: “The senescent phenotype is associated with an abnormal nuclear morphology which can be reminiscent of the p53-mediated cellular senescence pathway mediated by lamin A stabilisation (Yoon, Kang et al., 2019). However, we did not detect any significant change in Lamin A expression level upon H2A.Z.1 RNAi

9_My final comment relates to potential overlapping functions of H2AZ.1 and H2A.Z.2. Given the demonstrated importance for H2A.Z.2 in genome instability, it is surprising that the authors do not investigate any correlation between H2A.Z.2 and cancer progression/prognosis.
We do agree that it would be interesting but a thorough analyses of correlation with cancer and CIN and H2A.Z.2 would go beyond the scope of this manuscript. We added a comment on this point in the discussion.

10_ Also, on a related note, can the authors expand on why they believe high - as opposed to low, and thus comparable to the siRNA experiments - H2AFZ expression leads to worse outcomes/survival probability for cancer patients?
The new manuscript expands quite a lot on the significance of H2AFZ and MYC and I hope this will make the concept clear for the readership.

11_Also surprising is the finding that although H2AZ.1 specifically affects the expression of Aurora B, Borealin and Survivin, it is the H2A.Z.2 knockdown which displays a chromosome segregation phenotype. However, Figure 1 does show that micronuclei were observed in the H2AZ.1 RNAi, possibly indicating chromosome segregation defects. To address potential overlapping functions with H2A.Z.2, the authors should show whether Aurora B, Borealin and Survivin are correctly localised in the H2AZ.1 RNAi.
H2AZ.1 RNAi leads to a G1 arrest and senescence therefore the cells will never reach mitosis. Please refer to the Response to reviewer 2 point 5.

- Please correct the following typos:

Page 7, second paragraph - splice variants

Page 8, paragraph title should read 'sister chromatid cohesion'

Figure legend 4B should read H2A.Z.1 not H2A.Z.2

The typos indicated have been amended.

1st Revision - Editorial Decision 23rd Jul 2021

Dear Paola,

Thank you for submitting your revised manuscript. It has now been seen by two of the original referees.

As you can see, the referee finds that the study is significantly improved during revision and recommends publication. However, I need you to address the editorial points below before I can accept the manuscript.

• Please address the remaining minor concerns of referee #3.
• Please make the following data sets publicly available and remove the referee tokens from the manuscript:
http://www.ncbi.nlm.nih.gov/bioproject/629054
https://www.ncbi.nlm.nih.gov/geo/query/acc.cgi?acc=GSE173113
• Please provide 3-5 keywords for your study. These will be visible in the html version of the paper and on PubMed and will help increase the discoverability of your work.
• Please add a 'Conflict of Interests' section.
• As per our format requirements, in the reference list, citations should be listed in alphabetical order and then chronologically, with the authors' surnames and initials inverted; where there are more than 10 authors on a paper, 10 will be listed, followed by 'et al.'. The journal names should be italicized. Please see
https://www.embopress.org/page/journal/14693178/authorguide#referencesformat
• We note that the callouts of Fig EV2E,N+O and Fig EV3B+C are currently missing.
• The figure legends should be moved to after the Reference list. The tables should be at the end of the file.
• We note that there are two different Table 1s and 2s.
• Currently, your manuscript is in the format of a 'Research Article', which should have minimum 6 main figures as per our format requirements. Therefore, please convert one of the EV figures into a main figure. Please make sure to update the figure callouts in the text.
• Papers published in EMBO Reports include a 'synopsis' and 'bullet points' to further enhance discoverability. Both are displayed on the html version of the paper and are freely accessible to all readers. The synopsis includes a short standfirst summarizing the study in 1 or 2 sentences that summarize the paper and are provided by the authors and streamlined by the handling editor. I would therefore ask you to include your synopsis blurb and 3-5 bullet points listing the key experimental findings.
• In addition, please provide an image for the synopsis. This image should provide a rapid overview of the question addressed in the study but still needs to be kept fairly modest since the image size cannot exceed 550x400 pixels.

Thank you again for giving us to consider your manuscript for EMBO Reports, I look forward to your minor revision.

Kind regards,

Deniz

--
Deniz Senyilmaz Tiebe, PhD
Editor
EMBO Reports

Referee #1:

This ia a significantly revised manuscript by Sales Gill et al. The authors have added many new experiments and have clarified several aspects that within the manuscript. All of my previous concerns were addressed in a very satisfactory fashion.

Referee #3:

In this revised version of the manuscript, the authors have addressed most of my previous concerns.

The additional data on the loss of CENP-C and disruption to H3T3ph staining, which could potentially be linked to CPC mislocalisation, is particularly interesting and strengthens the proposed function for H2A.Z.2 in chromosome segregation. I also found the expanded discussion on the significance of H2AFZ and MYC expression useful and informative.

I have two remaining issues. The first is with respect to confirmation of H2A.Z.1 or H2A.Z.2 knockdown at the protein level. In the revised Figure EV1D the authors provide good evidence that each set of siRNA oligos is not effective on the other gene. However, this does not confirm knockdown of each at the protein level. This data could be easily added as a positive controls to this blot i.e. lanes showing GFP:H2A.Z.1 + H2A.Z.1 si and GFP:H2A.Z.2 + H2A.Z.2 si. Also, there appears to be a typo in the legend for this figure panel.

Second, I am still having difficulty interpreting the graph presented in Figure 3G. It is not clear to me whether all d2/d1 measurements for H2A.Z.1 (and not H2A.Z.2) were interpreted as significantly shifted towards the periphery? An additional explanation in the legend would probably help to clear this up.

To the Editorial Team
EMBO Reports

Dear Editor,

Thank you for your time in overseeing the revision process of our manuscript. We are pleased to know that the referees have found the study significantly improved and recommended its publication in EMBO Reports.
We are now ready to submit the final version of the paper that incorporates the editorial requests.

The remaining concerns of referee 3 have been addressed as follows:

1) The first is with respect to confirmation of H2A.Z.1 or H2A.Z.2 knockdown at the protein level. In the revised Figure EV1D the authors provide good evidence that each set of siRNA oligos is not effective on the other gene. However, this does not confirm knockdown of each at the protein level.
We have added to the panel the endogenous H2A.Z levels where it can clearly be seen that both knock downs (right two lanes) produce a reduction of the endogenous H2A.Z with a more significant depletion with H2A.Z.1 as expected by being the major form.

2)Also, there appears to be a typo in the legend for this figure panel.
We have corrected the typo.

3) I am still having difficulty interpreting the graph presented in Figure 3G. It is not clear to me whether all d2/d1 measurements for H2A.Z.1 (and not H2A.Z.2) were interpreted as significantly shifted towards the periphery? An additional explanation in the legend would probably help to clear this
We have added some more information in the figure legend as follows: The ratio between d2 and d1 gives the position of the centromere relative to the center of the nucleus. The graph represents the percentages of centromeres with distances following within the 5 binning categories.

2nd Revision - Editorial Decision 3rd Aug 2021

Dear Paola,

Thank you for submitting your revised manuscript. I have now looked at everything and all is fine. Therefore, I am very pleased to accept your manuscript for publication in EMBO Reports.

Congratulations on a nice work!

Kind regards,

Deniz
--
Deniz Senyilmaz Tiebe, PhD
Editor
EMBO Reports

At the end of this email I include important information about how to proceed. Please ensure that you take the time to read the information and complete and return the necessary forms to allow us to publish your manuscript as quickly as possible.

As part of the EMBO publication's Transparent Editorial Process, EMBO reports publishes online a Review Process File to accompany accepted manuscripts. As you are aware, this File will be published in conjunction with your paper and will include the referee reports, your point-by-point response and all pertinent correspondence relating to the manuscript.

If you do NOT want this File to be published, please inform the editorial office within 2 days, if you have not done so already, otherwise the File will be published by default [contact: emboreports@embo.org]. If you do opt out, the Review Process File link will point to the following statement: "No Review Process File is available with this article, as the authors have chosen not to make the review process public in this case."

Should you be planning a Press Release on your article, please get in contact with emboreports@wiley.com as early as possible, in order to coordinate publication and release dates.

Thank you again for your contribution to EMBO reports and congratulations on a successful publication. Please consider us again in the future for your most exciting work.

****

THINGS TO DO NOW:

You will receive proofs by e-mail approximately 2-3 weeks after all relevant files have been sent to our Production Office; you should return your corrections within 2 days of receiving the proofs.

Please inform us if there is likely to be any difficulty in reaching you at the above address at that time. Failure to meet our deadlines may result in a delay of publication, or publication without your corrections.

All further communications concerning your paper should quote reference number EMBOR-2020-52061V3 and be addressed to emboreports@wiley.com.

Should you be planning a Press Release on your article, please get in contact with emboreports@wiley.com as early as possible, in order to coordinate publication and release dates.

EMBO PRESS

YOU MUST COMPLETE ALL CELLS WITH A PINK BACKGROUND ➔

PLEASE NOTE THAT THIS CHECKLIST WILL BE PUBLISHED ALONGSIDE YOUR PAPER

Corresponding Author Name: Paola vagnarelli
Journal Submitted to: EMBO REPORTS
Manuscript Number: EMBOR-2020-52061V3

Reporting Checklist For Life Sciences Articles (Rev. June 2017)

This checklist is used to ensure good reporting standards and to improve the reproducibility of published results. These guidelines are consistent with the Principles and Guidelines for Reporting Preclinical Research issued by the NIH in 2014. Please follow the journal's authorship guidelines in preparing your manuscript.

A- Figures
1. Data
The data shown in figures should satisfy the following conditions:

- ➔ the data were obtained and processed according to the field's best practice and are presented to reflect the results of the experiments in an accurate and unbiased manner.
- ➔ figure panels include only data points, measurements or observations that can be compared to each other in a scientifically meaningful way.
- ➔ graphs include clearly labeled error bars for independent experiments and sample sizes. Unless justified, error bars should not be shown for technical replicates.
- ➔ if n< 5, the individual data points from each experiment should be plotted and any statistical test employed should be justified
- ➔ Source Data should be included to report the data underlying graphs. Please follow the guidelines set out in the author ship guidelines on Data Presentation.

2. Captions

Each figure caption should contain the following information, for each panel where they are relevant:

- ➔ a specification of the experimental system investigated (eg cell line, species name).
- ➔ the assay(s) and method(s) used to carry out the reported observations and measurements
- ➔ an explicit mention of the biological and chemical entity(ies) that are being measured.
- ➔ an explicit mention of the biological and chemical entity(ies) that are altered/varied/perturbed in a controlled manner.

- ➔ the exact sample size (n) for each experimental group/condition, given as a number, not a range;
- ➔ a description of the sample collection allowing the reader to understand whether the samples represent technical or biological replicates (including how many animals, litters, cultures, etc.).
- ➔ a statement of how many times the experiment shown was independently replicated in the laboratory.
- ➔ definitions of statistical methods and measures:
  - common tests, such as t-test (please specify whether paired vs. unpaired), simple χ2 tests, Wilcoxon and Mann-Whitney tests, can be unambiguously identified by name only, but more complex techniques should be described in the methods section;
  - are tests one-sided or two-sided?
  - are there adjustments for multiple comparisons?
  - exact statistical test results, e.g., P values = x but not P values < x;
  - definition of 'center values' as median or average;
  - definition of error bars as s.d. or s.e.m.

Any descriptions too long for the figure legend should be included in the methods section and/or with the source data.

In the pink boxes below, please ensure that the answers to the following questions are reported in the manuscript itself. Every question should be answered. If the question is not relevant to your research, please write NA (non applicable). We encourage you to include a specific subsection in the methods section for statistics, reagents, animal models and human subjects.

USEFUL LINKS FOR COMPLETING THIS FORM

http://www.antibodypedia.com
http://1degreebio.org
http://www.equator-network.org/reporting-guidelines/improving-bioscience-research-repo

http://grants.nih.gov/grants/olaw/olaw.htm
http://www.mrc.ac.uk/Ourresearch/Ethicsresearchguidance/Useofanimals/index.htm
http://ClinicalTrials.gov
http://www.consort-statement.org
http://www.consort-statement.org/checklists/view/32-consort/66-title
http://www.equator-network.org/reporting-guidelines/reporting-recommendations-for-tum
http://datadryad.org
http://figshare.com
http://www.ncbi.nlm.nih.gov/gap
http://www.ebi.ac.uk/ega
http://biomodels.net/
http://biomodels.net/miriam/
http://jjj.biochem.sun.ac.za
http://oba.od.nih.gov/biosecurity/biosecurity_documents.html
http://www.selectagents.gov/

B- Statistics and general methods

Please fill out these boxes ➔ (Do not worry if you cannot see all your text once you press return)

1.a. How was the sample size chosen to ensure adequate power to detect a pre-specified effect size?	To be able to reach a statistically significant test if a difference exist.
1.b. For animal studies, include a statement about sample size estimate even if no statistical methods were used.	NA
2. Describe inclusion/exclusion criteria if samples or animals were excluded from the analysis. Were the criteria pre-established?	NA
3. Were any steps taken to minimize the effects of subjective bias when allocating animals/samples to treatment (e.g. randomization procedure)? If yes, please describe.	Yes. Blind slides reading
For animal studies, include a statement about randomization even if no randomization was used.	NA
4.a. Were any steps taken to minimize the effects of subjective bias during group allocation or/and when assessing results (e.g. blinding of the investigator)? If yes please describe.	NA
4.b. For animal studies, include a statement about blinding even if no blinding was done	NA
5. For every figure, are statistical tests justified as appropriate?	YES
Do the data meet the assumptions of the tests (e.g., normal distribution)? Describe any methods used to assess it.	Test were choses based on the distribution of the data.

Is there an estimate of variation within each group of data?	All the data are reported either as scatter plots, violon plots or in bar plots.
Is the variance similar between the groups that are being statistically compared?	NA

C- Reagents

6. To show that antibodies were profiled for use in the system under study (assay and species), provide a citation, catalog number and/or clone number, supplementary information or reference to an antibody validation profile. e.g., Antibodypedia (see link list at top right), 1DegreeBio (see link list at top right).	
7. Identify the source of cell lines and report if they were recently authenticated (e.g., by STR profiling) and tested for mycoplasma contamination.	

* for all hyperlinks, please see the table at the top right of the document

D- Animal Models

8. Report species, strain, gender, age of animals and genetic modification status where applicable. Please detail housing and husbandry conditions and the source of animals.	NA
9. For experiments involving live vertebrates, include a statement of compliance with ethical regulations and identify the committee(s) approving the experiments.	NA
10. We recommend consulting the ARRIVE guidelines (see link list at top right) (PLoS Biol. 8(6), e1000412, 2010) to ensure that other relevant aspects of animal studies are adequately reported. See author guidelines, under 'Reporting Guidelines'. See also: NIH (see link list at top right) and MRC (see link list at top right) recommendations. Please confirm compliance.	NA

E- Human Subjects

11. Identify the committee(s) approving the study protocol.	NA
12. Include a statement confirming that informed consent was obtained from all subjects and that the experiments conformed to the principles set out in the WMA Declaration of Helsinki and the Department of Health and Human Services Belmont Report.	NA
13. For publication of patient photos, include a statement confirming that consent to publish was obtained.	NA
14. Report any restrictions on the availability (and/or on the use) of human data or samples.	NA
15. Report the clinical trial registration number (at ClinicalTrials.gov or equivalent), where applicable.	NA
16. For phase II and III randomized controlled trials, please refer to the CONSORT flow diagram (see link list at top right) and submit the CONSORT checklist (see link list at top right) with your submission. See author guidelines, under 'Reporting Guidelines'. Please confirm you have submitted this list.	NA
17. For tumor marker prognostic studies, we recommend that you follow the REMARK reporting guidelines (see link list at top right). See author guidelines, under 'Reporting Guidelines'. Please confirm you have followed these guidelines.	NA

F- Data Accessibility

18: Provide a "Data Availability" section at the end of the Materials & Methods, listing the accession codes for data generated in this study and deposited in a public database (e.g. RNA-Seq data: Gene Expression Omnibus GSE39462, Proteomics data: PRIDE PXD000208 etc.) Please refer to our author guidelines for 'Data Deposition'.<br><br>Data deposition in a public repository is mandatory for:<br>a. Protein, DNA and RNA sequences<br>b. Macromolecular structures<br>c. Crystallographic data for small molecules<br>d. Functional genomics data<br>e. Proteomics and molecular interactions	YES
19. Deposition is strongly recommended for any datasets that are central and integral to the study; please consider the journal's data policy. If no structured public repository exists for a given data type, we encourage the provision of datasets in the manuscript as a Supplementary Document (see author guidelines under 'Expanded View' or in unstructured repositories such as Dryad (see link list at top right) or Figshare (see link list at top right).	The data have been deposited
20. Access to human clinical and genomic datasets should be provided with as few restrictions as possible while respecting ethical obligations to the patients and relevant medical and legal issues. If practically possible and compatible with the individual consent agreement used in the study, such data should be deposited in one of the major public access-controlled repositories such as dbGAP (see link list at top right) or EGA (see link list at top right).	The data have been deposited
21. Computational models that are central and integral to a study should be shared without restrictions and provided in a machine-readable form. The relevant accession numbers or links should be provided. When possible, standardized format (SBML, CellML) should be used instead of scripts (e.g. MATLAB). Authors are strongly encouraged to follow the MIRIAM guidelines (see link list at top right) and deposit their model in a public database such as Biomodels (see link list at top right) or JWS Online (see link list at top right). If computer source code is provided with the paper, it should be deposited in a public repository or included in supplementary information.	NA

G- Dual use research of concern

22. Could your study fall under dual use research restrictions? Please check biosecurity documents (see link list at top right) and list of select agents and toxins (APHIS/CDC) (see link list at top right). According to our biosecurity guidelines, provide a statement only if it could.	04/08/202104/08/2021
